# Metabolic Glycoengineering Enables Fluorine-18 Radiolabeling of T Lymphocytes via Dual-Bioorthogonal Chemistry

**DOI:** 10.1021/acs.bioconjchem.6c00052

**Published:** 2026-04-24

**Authors:** Anisa Biti, Alessia Centanni, Surachet Imlimthan, Heidi Harjunpää, Arina Sukhova, Susanne K. Wiedmer, Susanna Fagerholm, Mirkka Sarparanta

**Affiliations:** † Department of Chemistry, Faculty of Science, 3835 University of Helsinki, 00560 Helsinki, Finland; ‡ Department of Molecular and Integrative Biosciences, Faculty of Biological and Environmental Sciences, University of Helsinki, 00790 Helsinki, Finland

## Abstract

The ability to track therapeutic cells is critical for advancing adoptive cell therapy. Positron emission tomography (PET) offers sensitive, quantitative imaging, but improved strategies for cell labeling remain needed. Here, we report a metabolic glycoengineering approach that installs azide groups onto the Jurkat T lymphocyte surface using the canonical tetraacetylated *N*-azidoacetylmannosamine (Ac_4_ManNAz). Azide-bearing cells were functionalized via strain-promoted azide–alkyne cycloaddition (SPAAC-based ligation) with a dual-clickable trancyclooctene (TCO)-bearing dibenzocyclooctyne (DBCO) derivative (sulfo-DBCO-PEG_4_-TCO), enabling subsequent inverse electron-demand Diels–Alder (IEDDA, TCO-tetrazine ligation) radiolabeling at cell–surface TCO moieties using an aluminum [^18^F]­fluoride-tetrazine (Al­[^18^F]­F-Tz) tracer. Labeling conditions were optimized to achieve suitable cell-associated activity while maintaining good viability. We evaluated Al­[^18^F]­F-Tz pharmacokinetics, in vitro fluorine-18-labeled Jurkat cells, and a proof-of-concept pretargeting strategy in athymic nude mice. In vitro fluorine-18-labeled cells exhibited predictable trafficking and biodistribution over the imaging period, supporting the feasibility of MGE-based bioorthogonal radiolabeling for PET cell tracking. In contrast, pretargeted imaging requires further optimization and was dominated by the hepatic and intestinal signal consistent with hepatobiliary clearance of Al­[^18^F]­F-Tz. Overall, these findings underscore the potential of MGE-mediated bioorthogonal radiolabeling as a nongenetic platform for PET tracking of immune cells and provide a foundation for further development of pretargeted approaches for adoptively transferred cells.

## Graphical abstract

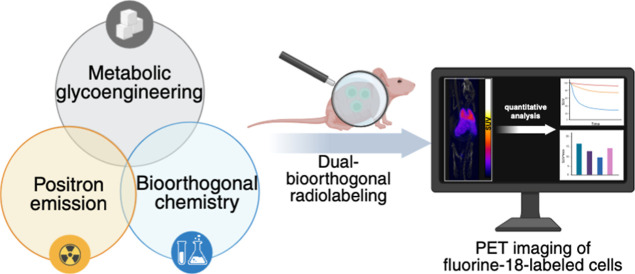

## Introduction

Adoptive cell therapy (ACT) harnesses autologous immune cells to recognize and destroy cancer cells and includes modalities such as chimeric antigen receptor T cells, T cell receptor (TCR)-engineered T cells, tumor-infiltrating lymphocytes, and natural killer (NK) cell therapies.^1^ While ACT has shown remarkable clinical success, noninvasive imaging to monitor the in vivo trafficking, persistence, and tumor infiltration of transferred cells remains critical for optimizing efficacy and safety.^2–4^

Molecular imaging technologies employed for noninvasive cell tracking include positron emission tomography (PET), single-photon emission computed tomography (SPECT), optical imaging, magnetic resonance imaging, magnetic particle imaging, and multimodal imaging. They differ in terms of spatial and temporal resolution, sensitivity, and depth of penetration of the signal. Among these, nuclear imaging techniques, particularly PET and SPECT, offer high sensitivity and quantitative accuracy for in vivo cell tracking. In favorable settings, PET/SPECT cell tracking has been reported with sensitivity approaching 1 × 10^5^ infused cells.^5^ PET cell tracking has been achieved using both direct (ex vivo) radiolabeling strategies that do not rely on genetic modification and indirect (in vivo) approaches based on reporter gene expression, which introduces additional regulatory complexity and potential immunogenicity, particularly for the already engineered cell products.^6^

Representative examples of direct PET cell labeling include the use of fluorine-18 (^18^F), copper-64, and zirconium-89-based tracers. For example, 2-deoxy-2-[^18^F]­fluoro-d-glucose ([^18^F]­FDG), the most widely used PET radiotracer in oncology, has been used to radiolabel macrophage-activated killer cells ex vivo for tracking in patients with metastatic ovarian carcinoma.^7^ Copper-64 has been used in combination with bioorthogonal chemistry to image metabolically glycoengineered ovalbumin-specific cytotoxic T lymphocytes (OVA-CTLs) in tumor-bearing mice.^8^ Longer-lived radionuclides such as zirconium-89, commonly used as the zirconium-89-oxine complex, have enabled monitoring of multiple immune cell types (e.g., CAR-T, γδ T, and NK cells) in murine and nonhuman primate models.^9–11^

Indirect PET cell labeling typically relies on engineered reporter gene expression, such as herpes simplex virus type 1 thymidine kinase (HSV1-tk), human sodium iodide symporter, or prostate-specific membrane antigen. For instance, HSV1-tk-expressing CAR-T cells have been imaged with 9-[4-[^18^F]­fluoro-3-(hydroxymethyl)­butyl]­guanine ([^18^F]­FHBG),^12^ while HSV1-tk-expressing Jurkat cells have been imaged with 2′-fluoro-2′-deoxy-1-β-d-arabinofuranosyl-5-[^124^I]­iodoracil ([^124^I]­FIAU).^13^ hNIS-expressing dendritic cells have been tracked with ^18^F-tetrafluoroborate ([^18^F]­TFB),^14^ and PSMA-expressing CAR-T cells with [^18^F]­DCFPyL.^15^

The physical half-life of the radionuclide imposes experimental and safety-related limitations. Because cellular migration and expansion can occur over several days, direct labeling with short-lived radionuclides such as fluorine-18 is generally limited to early trafficking studies, whereas long-lived radionuclides enable delayed imaging, albeit at the cost of increased radiation exposure.^16^ Pretargeted imaging can mitigate these trade-offs by decoupling the biological half-life of the targeting vector from the radionuclide physical half-life; the targeting vector is administered first and allowed to localize, followed by a radiolabeled small-molecule tracer that binds selectively to it.^17^ In principle, this can reduce the background signal and radiation dose while enabling the use of short-lived positron emitters. However, many reported pretargeted cell imaging approaches still rely on genetic modification or extensive ex vivo manipulation, which may limit their practical and translational potential.^17^

Metabolic glycoengineering (MGE) provides a nongenetic approach for the installation of reactive chemical handles on the cell surface through metabolic incorporation of synthetic carbohydrates (Figure 1A, left panel). These handles can subsequently selectively react with labeled tracers through bioorthogonal ligation reactions, defined as highly selective reactions that proceed in living systems without perturbing native biochemical processes.^18^ Azide-modified carbohydrates are widely used in MGE,^19,20^ and the strain-promoted azide–alkyne cycloaddition (SPAAC, Figure 1A, right panel) is among the most common reactions for subsequent functionalization (SPAAC-based ligation),^21,22^ with broad use in fluorescent imaging^23,24^ and targeted delivery.^21,25^ However, SPAAC-based ligation is often limited for radiolabeling at tracer concentrations by its comparatively slow kinetics [second-order rate constants (*k*) typically in the range 10^–3^ to 10^–1^ M^–1^ s^–1^, depending on the strained cyclic alkyne] and by nonspecific interactions associated with many DBCO-based reagents. In contrast, the inverse electron-demand Diels–Alder (IEDDA) reaction (Figure 1A, right panel) between a tetrazine (Tz) and *trans*-cyclooctene (TCO) (Tz-TCO ligation) proceeds with much faster kinetics (*k* ∼ 1–10^6^ M^–1^ s^–1^) and is well suited for pretargeted imaging, including applications using short-lived positron emitters such as fluorine-18.^26–28^

**Figure 1:**
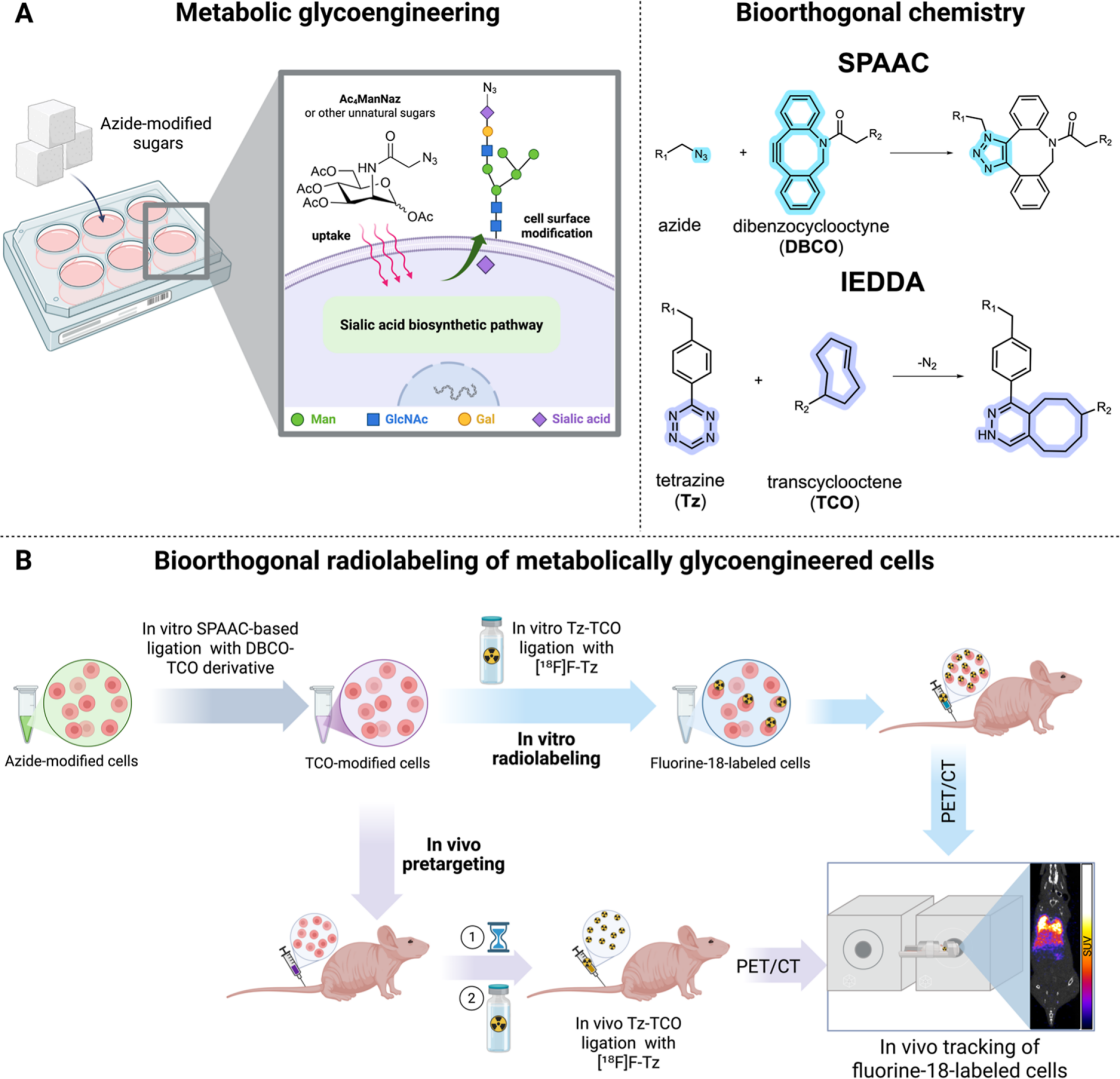
(A) Overview of MGE (left) and the bioorthogonal reaction schemes for SPAAC and IEDDA (right). (B) Schematic representation of the dual-bioorthogonal cell radiolabeling strategy mediated by MGE. Jurkat T cells are metabolically glycoengineered with Ac_4_ManNAz to introduce azide groups on cell–surface glycans, followed by SPAAC-based ligation of a sulfo-DBCO-PEG_4_-TCO derivative. The resulting cell–surface TCO moieties are then radiolabeled via IEDDA with a fluorine-18-labeled tetrazine (**[** ^**18**^ **F]­F-Tz**). Cells can be radiolabeled in vitro prior to administration for PET imaging or used in a pretargeting approach in which TCO-modified cells are injected first, allowed to distribute, and subsequently labeled in vivo by administration of **[** ^**18**^ **F]­F-Tz**. Created in BioRender. Biti, A. (2025) https://BioRender.com/8xdptas.

In this work, we metabolically glycoengineered Jurkat T cells with tetraacetylated *N*-azidoacetylmannosamine (Ac_4_ManNAz) to install azide groups on the cell surface, followed by SPAAC-based ligation using a hydrophilic TCO-bearing dibenzocyclooctyne linker (sulfo-DBCO-PEG_4_-TCO). This clickable intermediate enables subsequent Tz-TCO ligation with a fluorine-18-labeled Tz ([^18^F]­F-Tz) prosthetic group. The Tz was conjugated to the REstrained Complexing Agent (RESCA) chelator, enabling efficient radiolabeling via aluminum [^18^F]­fluoride (Al­[^18^F]­F) under mild conditions. Using this two-step approach, we assessed the feasibility of fluorine-18 radiolabeling of metabolically engineered T cells and explored its potential as a nongenetic strategy toward pretargeted PET imaging. A schematic overview of the approach is shown in Figure 1B.

## Results and Discussion

### Synthesis of Bioorthogonal Partners

The synthesis of sulfo-DBCO-PEG_4_-TCO (**1,** Scheme 1A) and radiolabeling precursor (+)-RESCA-Tz (**3,** Scheme 1B) was concise (2–3 steps) and straightforward. The sulfo-DBCO-PEG_4_ scaffold was chosen to improve the hydrophilicity relative to other commercially available DBCO derivatives, including the DBCO-PEG_2_-TCO initially evaluated in this study. Precursor **3** was designed to enable rapid ligation with TCO while allowing mild aluminum [^18^F]­fluoride radiolabeling. A hydrogen-substituted Tz was selected due to its superior reaction kinetics compared with Tzs bearing electron-donating substituents,^29^ as well as the commercial availability of NHS-activated derivatives suitable for conjugation. The RESCA chelator was originally developed to enable Al­[^18^F]F radiolabeling of sensitive biomolecules under mild conditions,^30^ which is advantageous for Tz-based radiotracers because tetrazines are susceptible to degradation under harsh radiolabeling conditions. To date, however, its application in small-molecule radiolabeling has remained relatively limited.^31–35^ We, therefore, investigated (+)-RESCA-mediated aluminum [^18^F]­fluoride radiolabeling as an alternative to established macrocyclic chelators such as NOTA (1,4,7-triazacyclononane-1,4,7-triacetic acid) and NODA (1,4,7-triazacyclononane-1,4-diacetic acid), which require elevated temperatures (100–120 °C) for efficient complexation.^36^ To the best of our knowledge, this work represents the first report of a (+)-RESCA-Tz conjugate. Given the acyclic structure of (+)-RESCA, the stability of the resulting complex warrants careful evaluation. Accordingly, precursor **3** was designed with a rigid linker between the (+)-RESCA chelator and the tetrazine moiety, which has been shown to improve the in vitro stability of the corresponding Al­[^18^F]­F-complex in our previous study.^35^

**Scheme 1:**
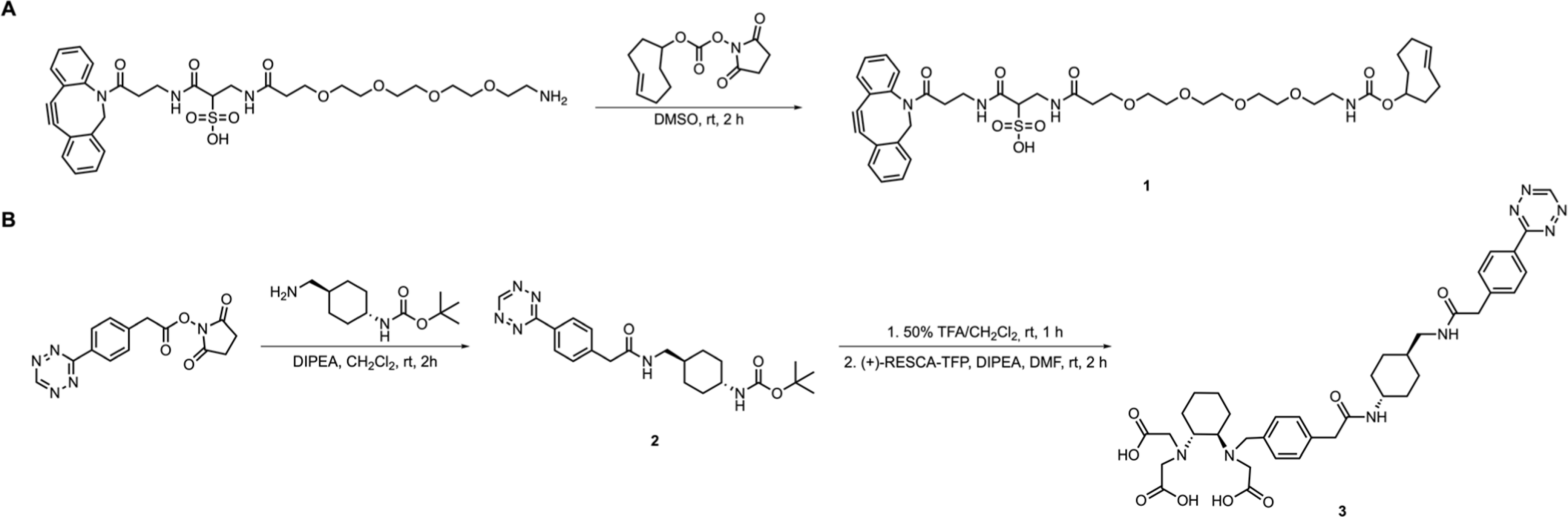
Synthesis of Bioorthogonal Partners Sulfo-DBCO-PEG_4_-TCO (**1**, A) and (+)-RESCA-Tz (**3**, B)

The target compounds were obtained in acceptable overall yields (48–50%) and were characterized by nuclear magnetic resonance (NMR) spectroscopy (^1^H NMR, ^13^C NMR, and 2D NMR, Figures S1–S7) and high-resolution mass spectrometry (HRMS, Figures S8–S9 and Table S1).

Furthermore, we investigated the formation of the nonradioactive reference complex **Al** ^**nat**^ **F-3** (where ^nat^F denotes nonradioactive fluorine with the natural isotopic composition, Scheme S1); the high-performance liquid chromatography (HPLC) and ^19^F NMR analysis are provided in Figure S10.

### Radiosynthesis of **Al­[** ^**18**^ **F]­F-3**

In this work, we used the enantiomerically pure (+)-RESCA due to its commercial availability and the absence of evidence that the racemic mixture offers superior chelation efficiency or improved Al­[^18^F]F complex stability.

The optimized radiosynthesis conditions (Scheme 2) were established in our previous study.^35^ The radiochemical conversion (RCC; 94.7 ± 4.6%, *n* = 38) was determined by radio-instant TLC (radio-iTLC) and was robust across the tested range of starting activity (232–1243 MBq) and reaction volume (370–940 μL), with no apparent dependence on either parameter (Figure 2A). Following Alumina N cartridge purification, **Al­[** ^**18**^ **F]­F-3** was obtained with radiochemical purity (RCP) > 98%. RCC and RCP were determined only by radio-TLC (examples in Figures S11 and S12) because [^18^F]­F^–^ can be retained on a reversed-phase HPLC column, potentially leading to overestimation of the RCC by radio-HPLC.^37,38^ Radio-HPLC was therefore used only to confirm the retention time and to verify the absence of additional fluorine-18-labeled species in quality control (QC) and stability samples.

**Scheme 2:**
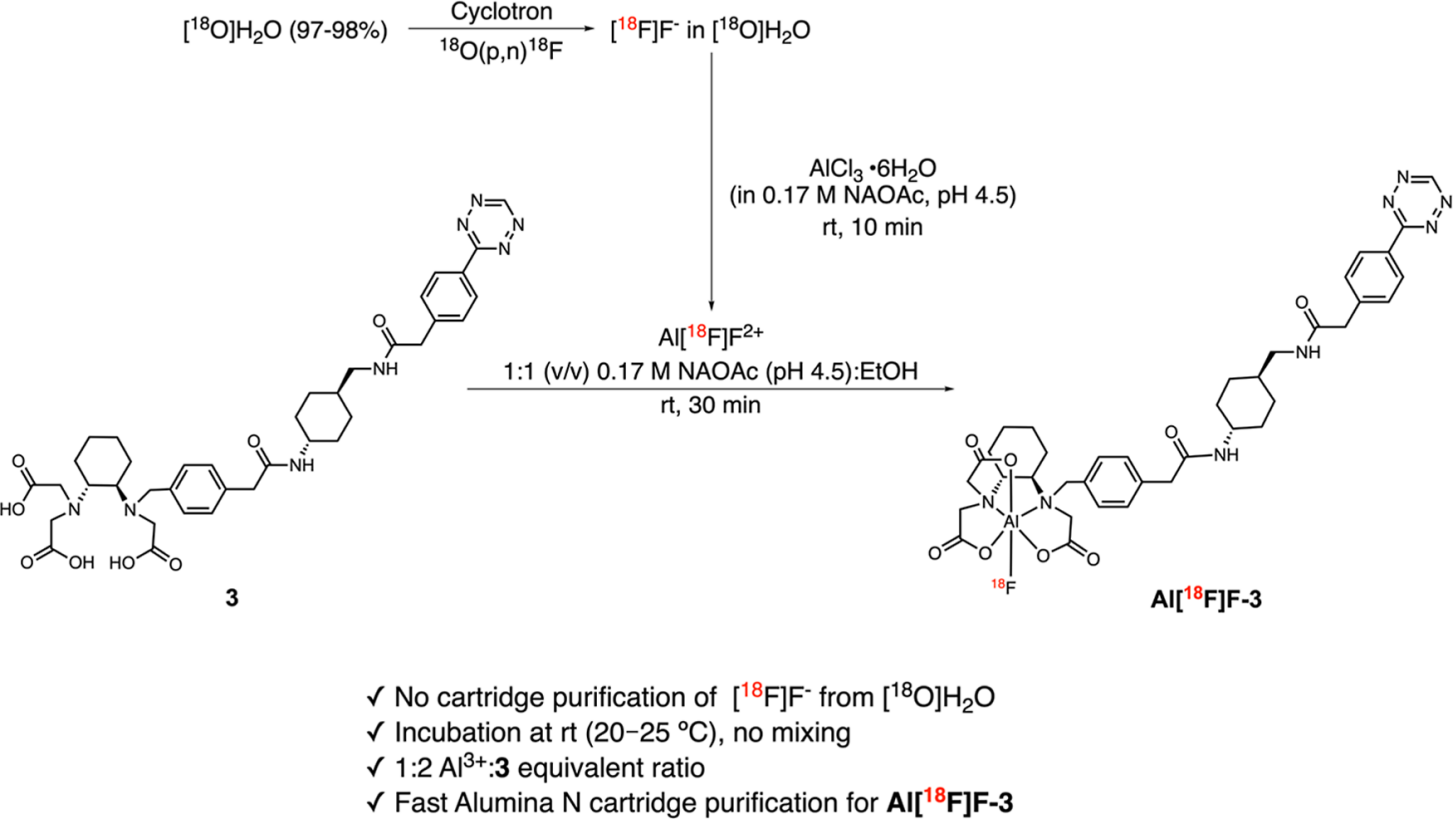
Radiolabeling of Precursor **3** Using the Al­[^18^F]F Method and Optimized Conditions

**Figure 2:**
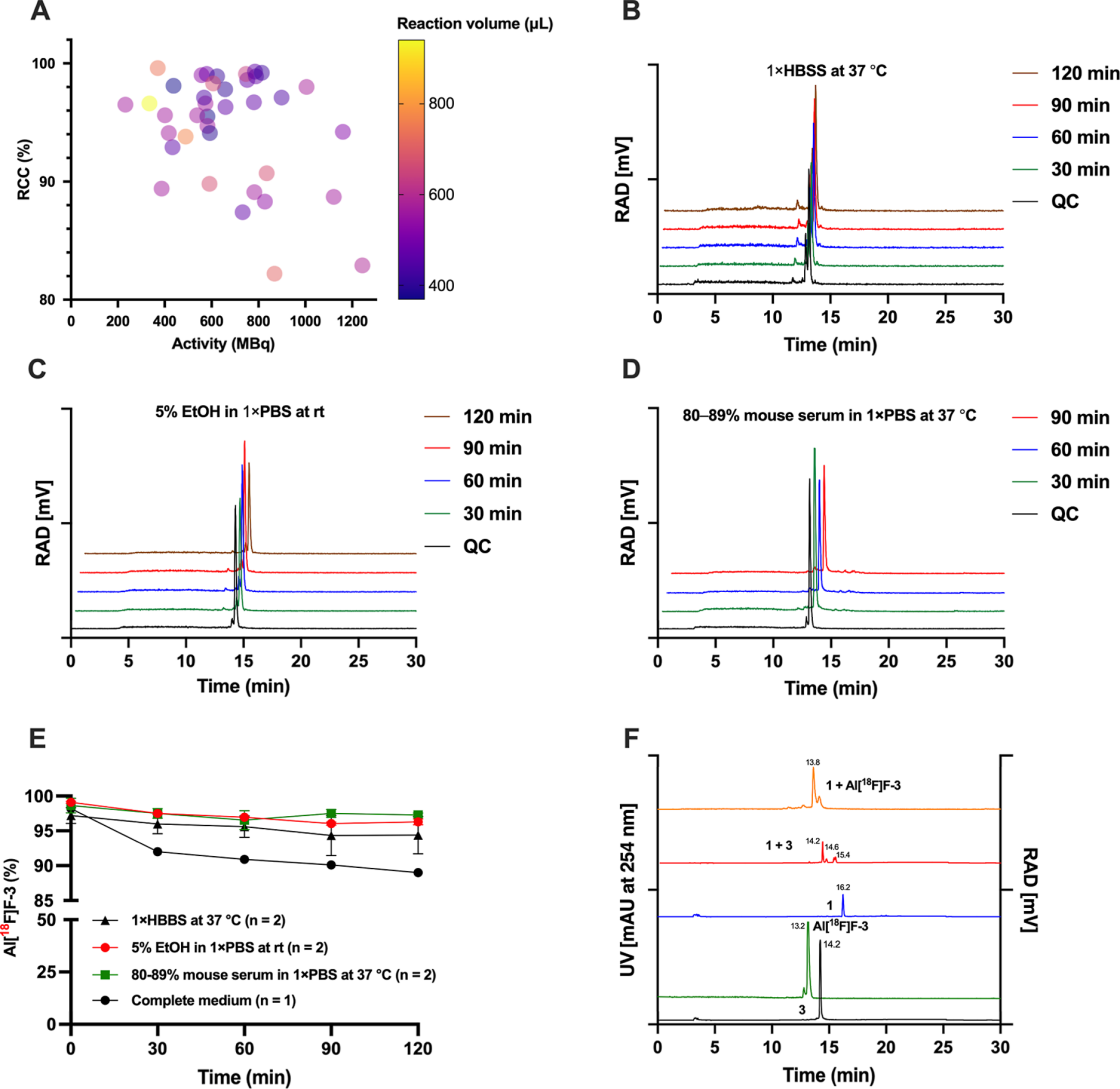
(A) Radiochemical conversion (RCC, *n* = 38) of **Al­[** ^**18**^ **F]­F-3** as a function of starting activity (*x* axis) and reaction volume (color-coded symbols). (B–D) Representative radio-HPLC chromatograms illustrating the radiolabel stability of **Al­[** ^**18**^ **F]­F-3** in different media. The QC (0 min) sample is shown in black (retention time 14.2 min), and stability samples collected at predetermined time points are shown in color. (E) Fraction of intact **Al­[** ^**18**^ **F]­F-3** (%) in the QC (0 min) and at indicated time points under the tested conditions, determined by radio-TLC. **F**. HPLC analysis of reference compounds **1** and **3**, **Al­[** ^**18**^ **F]­F-3,** and IEDDA reaction mixtures. UV chromatograms (left *y*-axis) show compounds **1** (16.2 min, blue), **3** (14.2 min, black), and their reaction mixture after 30 min, revealing residual **3** at 14.2 min and new peaks at 14.8 and 15.4 min (red). Radio-HPLC chromatograms (right *y*-axis) show **Al­[** ^**18**^ **F]­F-3 (**green) and the IEDDA reaction mixture of **1** with **Al­[** ^**18**^ **F]­F-3** after 30 min (orange), with the new peak at 13.8 min.

**Figure 3:**
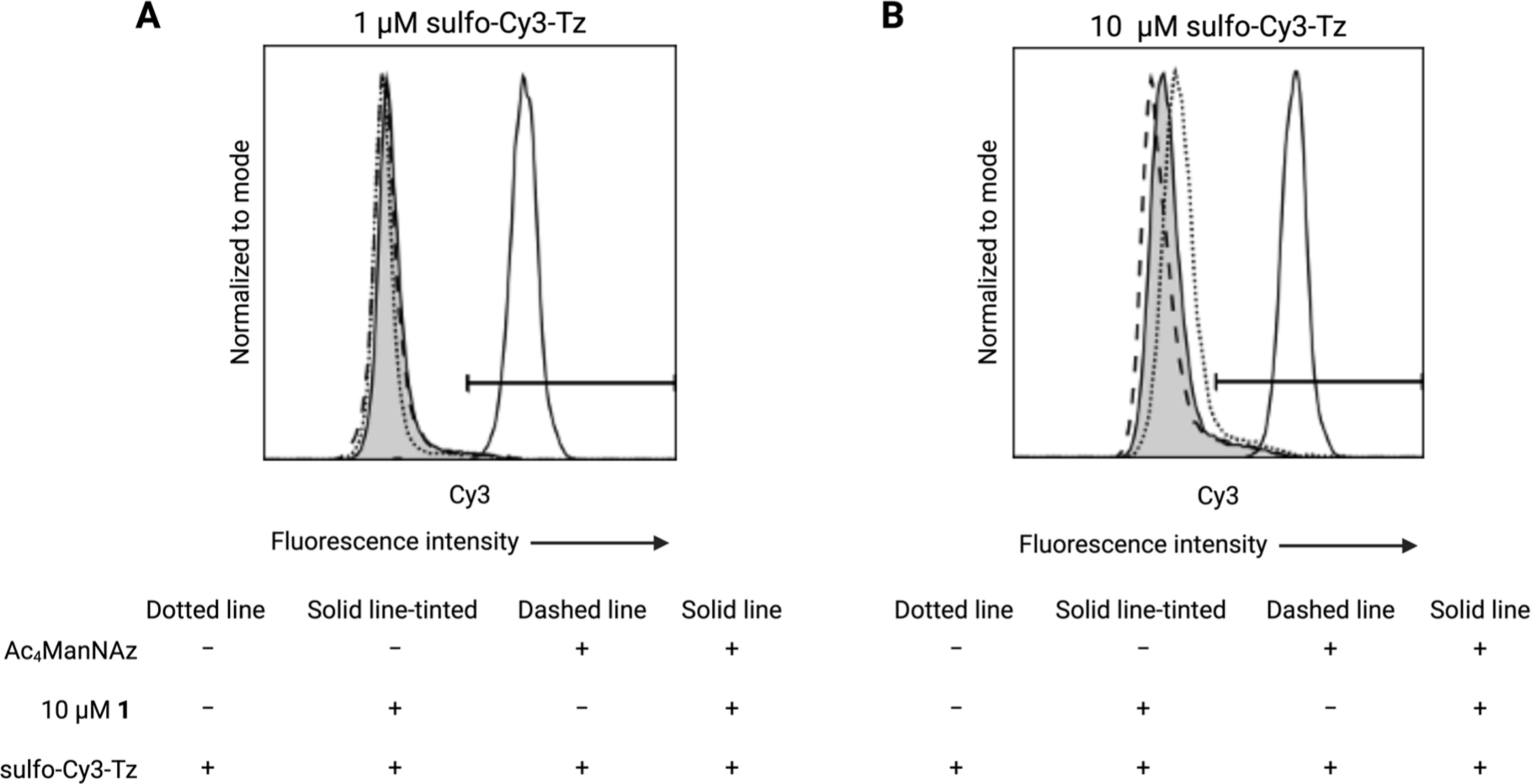
Flow cytometry analysis of two-step cell–surface labeling. (A) Labeling with 1 μM sulfo-Cy3-Tz. (B) Labeling with 10 μM sulfo-Cy3-Tz. Jurkat cells were treated with 0.5% v/v DMSO (control) or Ac_4_ManNAz for 72 h and incubated with sulfo-Cy3-Tz alone (dotted lines), with compound **1** followed by sulfo-Cy3-Tz (solid tinted lines), or treated with Ac_4_ManNAz and incubated with sulfo-Cy3-Tz (dashed lines) or with compound **1** followed by sulfo-Cy3-Tz (solid lines). Minimal fluorescence was observed in the absence of compound **1** in both control and Ac_4_ManNAz-treated cells. In contrast, efficient labeling was achieved in Ac_4_ManNAz-treated cells following SPAAC-based ligation with compound **1** and subsequent Tz-TCO ligation. Histograms are representative examples from a single sample per condition.

### In Vitro Evaluation of **Al­[** ^**18**^ **F]­F-3**: Stability, Lipophilicity, and Apparent Molar Activity

The radiolabel stability of **Al­[** ^**18**^ **F]­F-3** was initially assessed at 37 °C in 1 × Hanks’ balanced salt solution (HBSS, pH 7.2, *n* = 2), 80–89% mouse serum (*n* = 2), 5% EtOH in 1 × phosphate-buffered saline (PBS, pH 7.4, formulation) at room temperature (rt = 20–25 °C; *n* = 2), and complete cell culture medium [RPMI 1640 supplemented with 10% fetal bovine serum, 1% penicillin–streptomycin (PS), and 1% glutamine, *n* = 1]. Radio-HPLC analysis across these conditions (Figure 2B–D and stability in complete cell medium in Figure S13A) showed no evidence of additional fluorine-18-labeled species. Radio-TLC was used to determine defluorination and/or demetalation over time (Figure 2E). The degree of defluorination and/or demetalation remained <5% over 120 min under all tested conditions, except in complete cell culture medium, where the intact fraction of **Al­[** ^**18**^ **F]­F-3** decreased from 98.2% (QC sample) to 89% at 120 min.

Prompted by our attempts to synthesize and isolate **Al** ^**nat**^ **F-3**, which revealed decomplexation of the fluorine under the acidic HPLC conditions (Figure S10A), we carried out additional stability tests under acidic HPLC-like conditions (50:50 *v*/*v*, 0.1% trifluoroacetic acid in mQ: acetonitrile (pH 2–2.5) at rt (*n* = 2) and 40 °C (*n* = 2). As expected, radio-TLC indicated pronounced instability under these conditions, with heating greatly accelerating decomplexation of fluorine-18 (Figure S13B). The intact fraction of **Al­[** ^**18**^ **F]­F-3** decreased by 17.6 ± 0.99% at rt and 75 ± 0.35% at 40 °C. These findings further support the use of radio-TLC (rather than radio-HPLC) for RCC determination when investigating Al­[^18^F]­F-(+)-RESCA complexes.

**Al­[** ^**18**^ **F]­F-3** exhibited moderate lipophilicity (LogD_pH7.4_ = −2.37 ± 0.06, *n* = 2). This behavior is consistent with the overall polarity of the RESCA molecule while reflecting contributions from lipophilic motifs in the chelator and linker (including *trans*-cyclohexyl and the phenyl building blocks).^38^ Al­[^18^F]­F-RESCA and Al^nat^F-RESCA complexes have been reported to carry a net negative charge,^38^ although the charge was not independently assessed here.

The apparent molar activity (apparent *A*_m_ = 9.22 ± 4.44 GBq/μmol, *n* = 4) was estimated from the 534 nm HPLC traces of both precursor **3** and **Al­[** ^**18**^ **F]­F-3**. Under these analytical conditions, the measured precursor signal reflects both residual and potentially decomplexed precursors; therefore, the value reported is an apparent *A*_m_.

### In Vitro Bioorthogonal Ligations

Compound **1** reacted readily with Ac_4_ManNAz upon incubation at 37 °C in 1 × PBS (pH 7.4, Scheme S2A), as confirmed by HPLC analysis (Figure S14). After 60 min, only minor residual traces of **1** remained, and two major new peaks were observed, consistent with the formation of separable triazole regioisomers commonly produced in SPAAC-based ligations.^39,40^

Tz-TCO ligations between **1** and **3,** or between **1** and **Al­[** ^**18**^ **F]­F-3**, in 1 × PBS (pH 7.4, Scheme S2B) at 37 °C also proceeded rapidly. HPLC chromatograms are shown in Figure 2F (red and orange traces, respectively), and LC–MS confirmation for the nonradioactive reaction is provided in Figures S15–S16. In the nonradioactive reaction (red trace), **1** was fully consumed within 30 min, while a residual amount of **3** remained. Two new peaks were formed, and LC–MS analysis confirmed that one corresponded to the expected IEDDA adduct. In the radioactive reaction, **Al­[** ^**18**^ **F]­F-3** was completely consumed after 30 min, indicating that Tz reactivity is retained after radiolabeling. No further optimization of these in vitro reactions was conducted, as the conditions do not fully recapitulate the kinetics and steric environment encountered when azide or TCO groups are present on the cell surface.

### In Vitro Cell Studies

#### Flow Cytometry

Jurkat cells—an immortalized human T lymphocyte line originally derived from an acute T-cell leukemia patient—were selected as the model system due to their extensive characterization and widespread use in immuno-oncology research.^41^ Meanwhile, Ac_4_ManNAz is routinely used for MGE across diverse cell types, including Jurkat cells.^42^

The efficiency of MGE and subsequent TCO modification via SPAAC was first evaluated by flow cytometry using a fluorescent sulfo-cyanine3-tetrazine probe (sulfo-Cy3-Tz, Lumiprobe, Westminster, MD, USA) for IEDDA reactivity. Initial experiments with a commercially available DBCO-PEG_2_-TCO derivative are described in the Supporting Information (Figure S17 and Table S2). These studies revealed substantial nonspecific membrane association of DBCO-PEG_2_-TCO, which motivated the synthesis and use of the more hydrophilic compound **1** used in this work.

Incubation with compound **1** followed by sulfo-Cy3-Tz produced little to no labeling in the control cells, indicating minimal nonspecific membrane binding. In Ac_4_ManNAz-treated cells, however, fluorescence increased substantially, reaching >99% Cy3-positive cells with markedly increased median fluorescence intensity (MFI) values (Table S3). Although the fraction of Cy3-positive cells was similar at 1 and 10 μM of sulfo-Cy3-Tz (Figure 3A,B, respectively), the MFI at 10 μM was nearly 2-fold higher, consistent with more efficient Tz-TCO ligation at a higher Tz concentration.

Together, these results show that (i) Ac_4_ManNAz-mediated MGE is necessary for efficient SPAAC-based installation of TCO handles, (ii) the reduced lipophilicity of compound **1** relative to DBCO-PEG_2_-TCO substantially decreases nonspecific membrane association, and (iii) sulfo-Cy3-Tz alone produces a negligible background signal. Collectively, these findings support the use of compound **1** as a clickable intermediate for the two-step SPAAC–IEDDA labeling of azide-modified cells.

### In Vitro Cell Radiolabeling

Following initial radiolabeling experiments investigating incubation conditions, washing procedures, and tracer activity (experimental details in Supporting Information, Figure S18 and Table S4, results in Figure S19A–D), we implemented the optimized protocol summarized in Figure 4. Using this protocol, we further evaluated how the initial cell number and tracer activity influenced the radiolabeling performance (Figure 5A–D).

**Figure 4:**
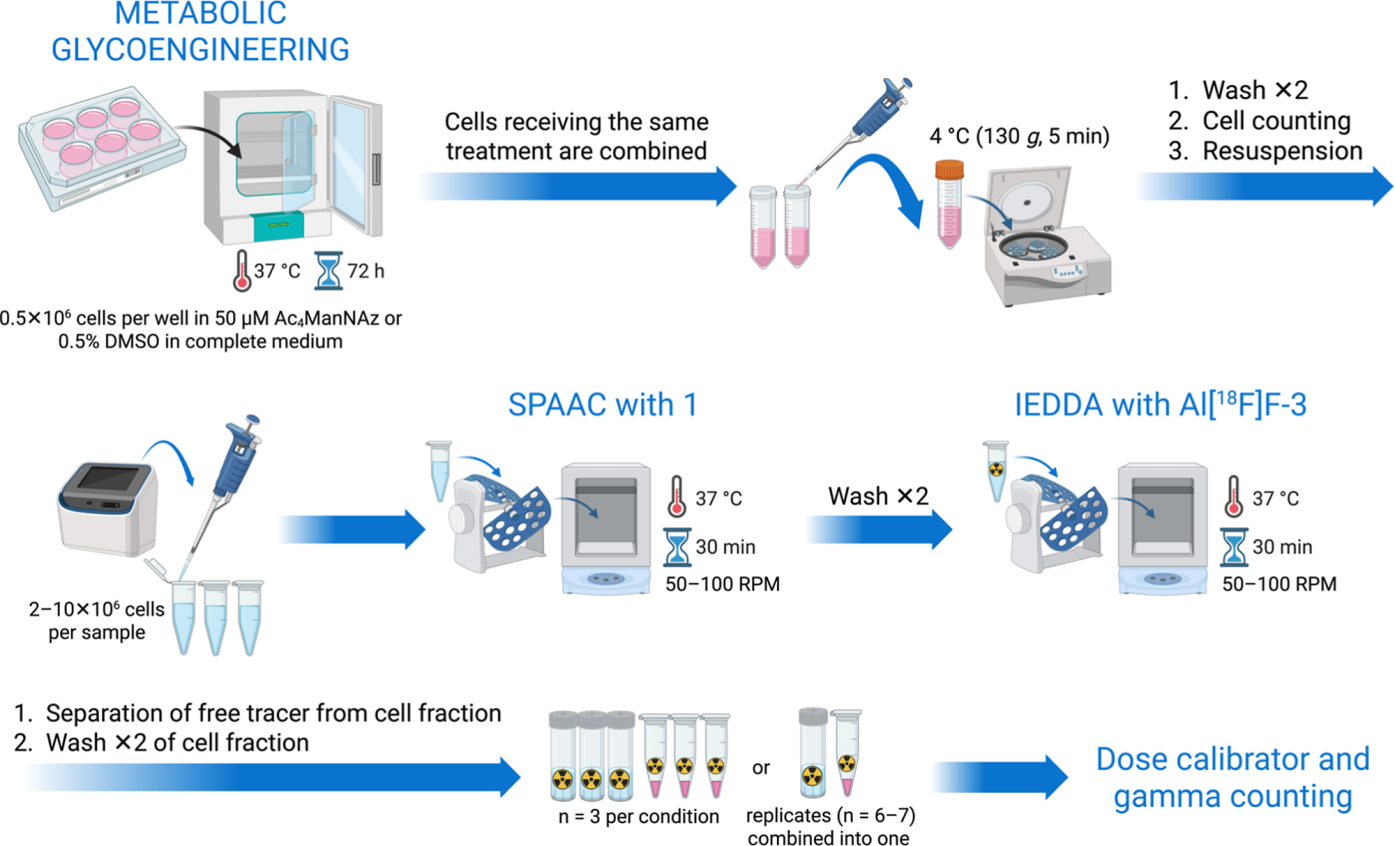
Schematic overview of the MGE-based cell radiolabeling protocol. Jurkat cells were metabolically glycoengineered by incubation with 50 μM Ac_4_ManNAz or 0.5% v/v DMSO (control) in complete medium for 72 h at 37 °C. Cells from wells receiving the same treatment were combined after MGE, washed twice, counted, and resuspended to obtain 2 × 10^6^ to 10 × 10^6^ cells per sample for SPAAC-based ligation with compound **1** at 37 °C for 30 min, followed by two washes. Cells were subsequently incubated with **Al­[** ^**18**^ **F]­F-3** for Tz-TCO ligation at 37 °C for 60 min, followed by two washes. Final cell pellets and free fractions were measured using a dose calibrator and gamma counting. Created in BioRender. Biti, A. (2026) https://BioRender.com/pzxzkp5.

**Figure 5:**
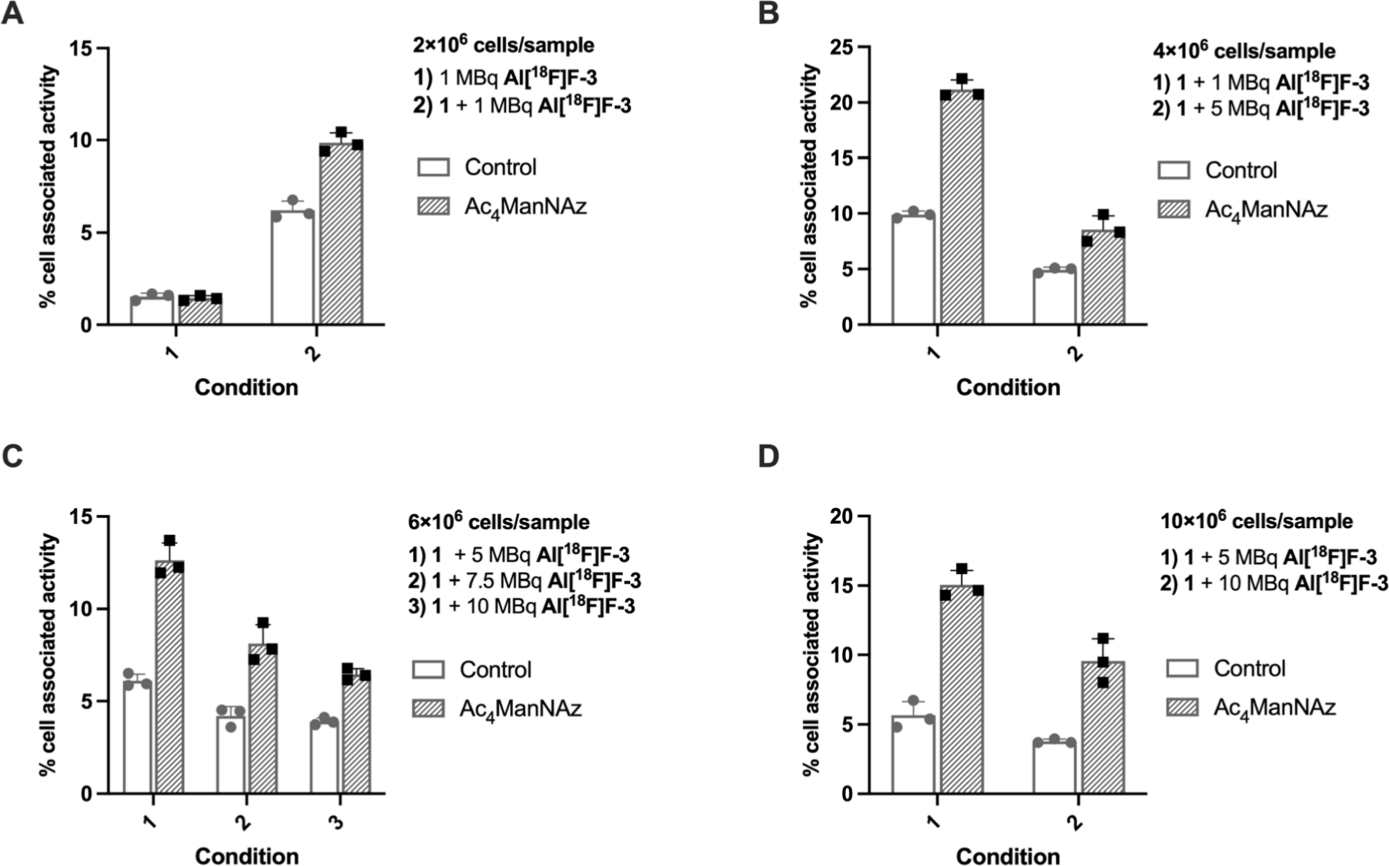
Cell-associated activity (%) of control and metabolically glycoengineered Jurkat cells following SPAAC-based and Tz-TCO ligations under different conditions (A–D). Influence of the cell number (2–10 × 10^6^) and tracer activity (1–10 MBq) on the radiolabeling efficiency when SPAAC-based ligation was done with 10 μM of compound **1** at 37 °C for 60 min. Results are presented as mean ± SD (*n* = 3 technical replicates).

Across all tested conditions, Ac_4_ManNAz-treated cells exhibited higher cell-associated activity than control cells, confirming successful azide installation by MGE and subsequent labeling via Tz-TCO ligation. Increasing the starting cell number (from 2 × 10^6^ to 10 × 10^6^) during the incubation with compound **1** generally reduced the cell-associated activity, indicating that labeling efficiency depends in part on the cell-to-activity ratio and highlighting the need to balance the cell number with the available radiotracer. For a given cell number, increasing the starting activity of **Al­[** ^**18**^ **F]­F-3** did not lead to a proportional increase in the labeling efficiency; instead, the labeling efficiency decreased slightly at higher activities (Figure 5A–D). This indicates a suboptimal Tz/TCO ratio at higher tracer concentrations, which may compromise the yield of the Tz-TCO ligation. Overall, the best labeling performance was seen at intermediate starting cell number (4–6 × 10^6^ cells/sample) and moderate activity (1–5 MBq/sample).

Viable cell numbers and viability were measured for samples presenting the highest cell-associated activity. With 4 × 10^6^ cells/sample (Figure 5B), the maximum cell-associated activity was 0.245 MBq, corresponding to ∼1.3 × 10^6^ viable cells (89%). With 6 × 10^6^ cells (Figure 5C), the maximum reached 0.413 MBq for ∼3.2 × 10^6^ viable cells (82%), and with 10 × 10^6^ cells (Figure 5D), 0.811 MBq was obtained for ∼3.4 × 10^6^ viable cells (81%). These results indicate a substantial loss of viable cells over the course of the ligation incubations and subsequent washing steps. Radiolabeling results were normalized to the added activity of **Al­[** ^**18**^ **F]­F-3** using counting standards.

To quantify nonspecific background binding in the experiments shown in Figure 5A–D (Table S5, entries **a**–**d**), empty microtubes were processed in parallel with compound **1** and/or **Al­[** ^**18**^ **F]­F-3**. Microtubes exposed only to 1 MBq of **Al­[** ^**18**^ **F]­F-3** showed negligible associated activity (0.1 ± 0.1%), confirming minimal adsorption of the radiotracer to plastic. In contrast, microtubes treated with both compound **1** and **Al­[** ^**18**^ **F]­F-3** exhibited considerably higher associated activity (3.1 ± 0.1% to 5.0 ± 0.8% at 1 MBq and 1.0 ± 0.2% at 5 MBq of **Al­[** ^**18**^ **F]­F-3**), consistent with adsorption of compound **1** to the plastic surface followed by reaction with **Al­[** ^**18**^ **F]­F-3**. This background contributes to the overestimation of true cell-associated activity in the analysis and underscores the importance of minimizing contact with reactive surfaces and ensuring efficient separation of cells during radiolabeling workflows.

Balancing background signal, labeling efficiency, cell loss during processing, and the need to obtain sufficient activity for in vivo studies, we next explored radiolabeling across multiple microtubes, followed by pooling of labeled cells into a single tube immediately before the final washing steps. This also allowed for the assessment of viable cell number and viability from a single sample. Incubation of 4 × 10^6^ cells with 10 MBq per tube (*n* = 6) yielded 0.472 MBq associated activity, corresponding to ∼6.2 × 10^6^ viable cells (80% viability). In a second experiment, incubation of 4 × 10^6^ cells with 7 MBq per tube (*n* = 7) yielded 0.864 MBq, corresponding to ∼10 × 10^6^ viable cells (76% viability). Based on these results, radiolabeling of 4 × 10^6^ cells with 7 MBq per tube was selected for the preparation of fluorine-18-labeled cells for animal studies.

A higher apparent labeling degree was also observed in exploratory experiments using filter-based separation after the IEDDA step (Figure S19E,F). However, due to concerns that the filtration could adversely affect cell viability, this approach was not pursued further (experimental details are in the Supporting Information).

### Animal Studies

**Al­[** ^**18**^ **F]­F-3**, in vitro fluorine-18-labeled cells, and pretargeted imaging of TCO-modified cells (with Ac_4_ManNAz-treated cells lacking TCO serving as a control) were evaluated in female athymic nude mice by dynamic PET/CT imaging and ex vivo biodistribution (Figure 6A). Summed PET images were reconstructed over predetermined intervals (0–15, 15–30, 30–60, and 0–90 min, Figure 6B–F), with corresponding grayscale images provided in Figure S20. Time-activity curves (TACs) and area-under-the-curve (AUC) analyses are shown in Figures 7A,B and S21, with statistical analysis of TACs summarized in Table S6. Ex vivo biodistribution in selected organs is presented in Figures 7C and S22 (all organs) and Figure S23 (selected organs per condition) and Table S7 (values). Additionally, dynamic PET/CT movies for each condition are provided in Supporting Information Movies S1–S5.

**Figure 6:**
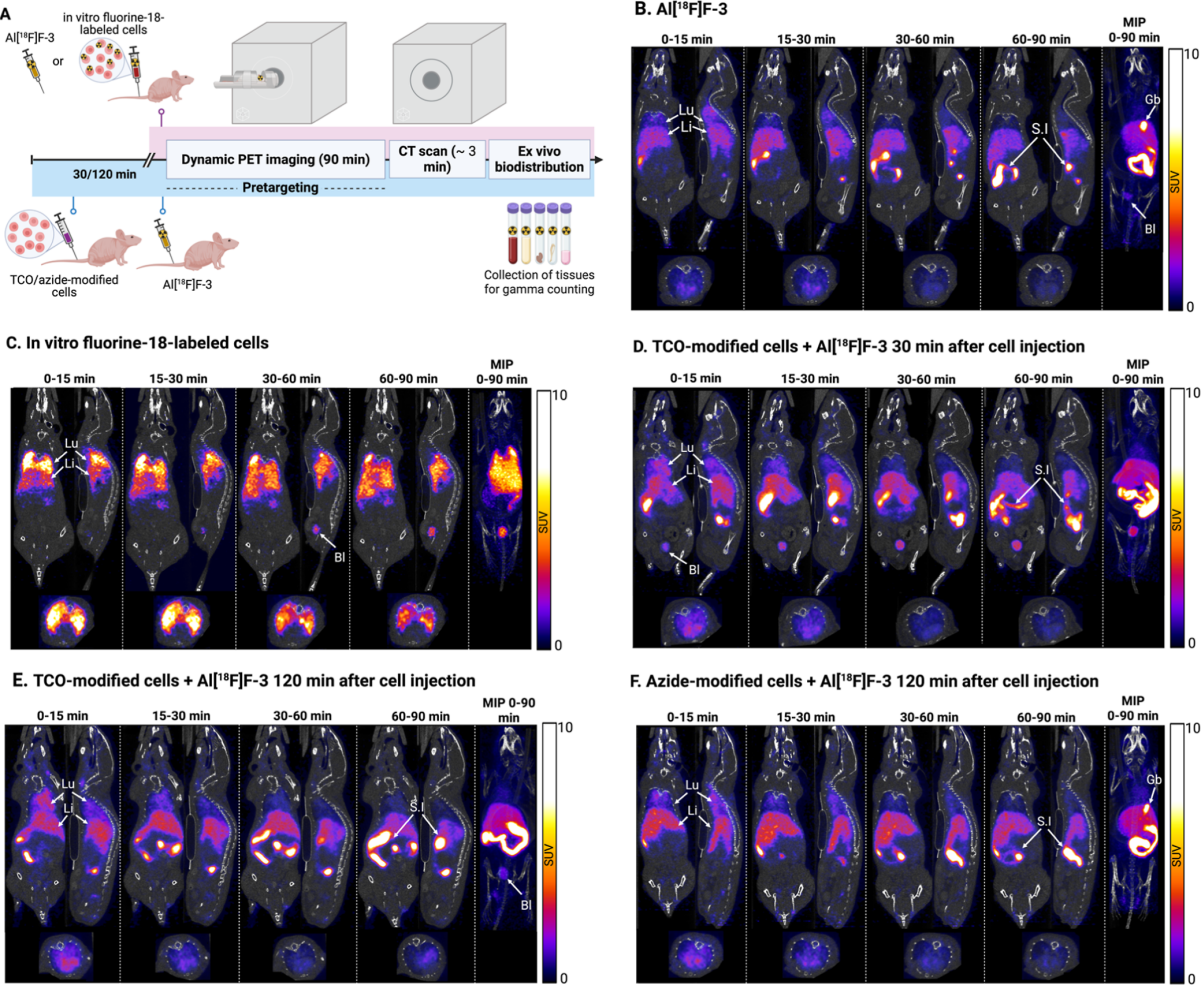
(A) Schematic representation of the study design for in vivo evaluation of the cell radiolabeling strategy. Created in BioRender. Biti, A. (2025) https://BioRender.com/pb6sgon (B–F). Representative summed PET/CT images (0–15, 15–30, 30–60, and 0–90 min) in coronal (left), sagittal (right), and transverse (bottom, through the lungs) planes, together with a maximum intensity projection (MIP, 0–90 min) following intravenous injection of **Al­[** ^**18**^ **F]­F-3** (B), in vitro fluorine-18-labeled cells (C), TCO-modified cells and **Al­[** ^**18**^ **F]­F-3** after 30 min (D), TCO-modified cells and **Al­[** ^**18**^ **F]­F-3** after 120 min (E), and azide-modified cells and by **Al­[** ^**18**^ **F]­F-3** after 120 min. Lu denotes lungs, Li denotes the liver, S.I denotes the small intestine, Gb denotes the gallbladder, and Bl denotes the bladder. The corresponding grayscale PET images without CT overlay are provided in Figure S20.

**Figure 7:**
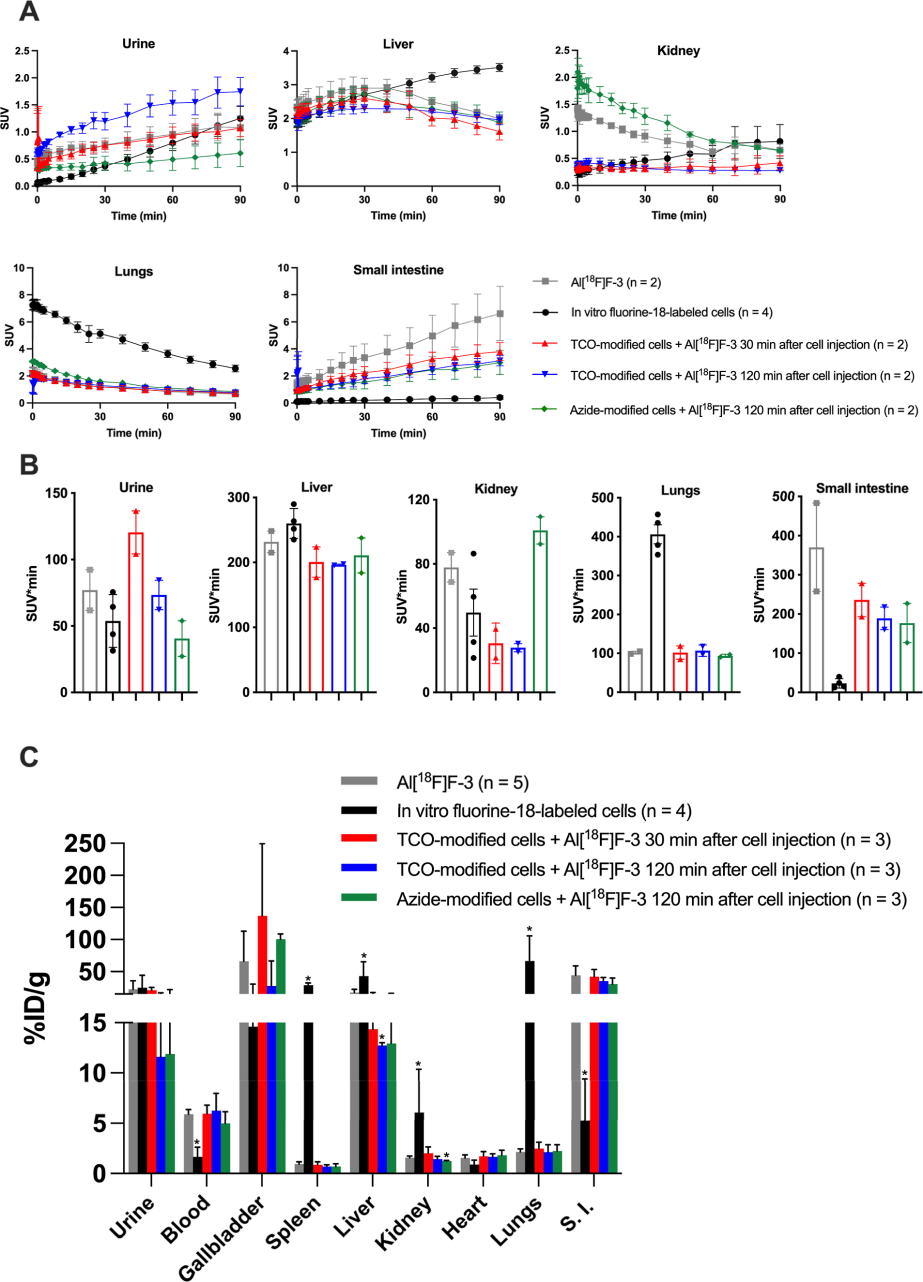
Quantitative PET analysis and ex vivo biodistribution of **Al­[** ^**18**^ **F]­F-3,** in vitro fluorine-18-labeled cells, and pretargeting conditions (TCO-modified cells, followed by **Al­[** ^**18**^ **F]­F-3** injection after 30 min, TCO-modified cells, followed by **Al­[** ^**18**^ **F]­F-3** injection after 120 min, and azide-modified cells, followed by **Al­[** ^**18**^ **F]­F-3** injection after 120 min). (A) TACs from dynamic PET imaging for selected ROIs. Data points correspond to mean ± SEM (*n* = 2). Comparisons with the control **Al­[** ^**18**^ **F]­F-3** were done using two-way ANOVA with Šídák’s multiple comparisons test. (B) Area-under-the-curve (AUC) analysis for selected ROIs. Error bars indicate SEM (C). Ex vivo biodistribution after the 90 min dynamic PET/CT scan, reported as percent injected dose per gram of tissue (%ID/g, mean ± SD, *n* = 2–5). Comparisons of the control **Al­[** ^**18**^ **F]­F-3** for (B,C) were done using the Mann–Whitney *U* test, and the results of the analysis are shown only when there is a statistical difference; the significance was set at *p < 0.05, **p < 0.01, and ***p < 0.001.

Following intravenous (iv) administration, **Al­[** ^**18**^ **F]­F-3** cleared rapidly from the blood pool, with prominent liver uptake evident within the first 15 min (Figure 6B). Over time, activity progressively accumulated in the liver and small intestine, and the 0–90 min MIP also revealed gallbladder accumulation. Ex vivo biodistribution at 90 min corroborated these findings (Figure 7C), indicating predominantly hepatobiliary clearance of **Al­[** ^**18**^ **F]­F-3**. The highest uptake values after imaging were observed in the gallbladder (66.19 ± 6.59% ID/g), small intestine (44.47 ± 4.52% ID/g), and liver (17.16 ± 5.53% ID/g). This profile is in accordance with the reported biodistribution of Al­[^18^F]­F-RESCA and other RESCA-based small molecules.^32,34,38^ The hepatobiliary route is plausibly influenced by lipophilic elements within the chelator framework (e.g., *trans*-cyclohexyl block and phenyl ring), which may be further reinforced by the additional *trans*-cyclohexyl unit in the linker. However, high activity was also detected in the urine (22.63 ± 13.1% ID/g), along with modest bone uptake (tibia 2.08 ± 0.74% ID/g and occipital 1.90 ± 0.6% ID/g), suggesting a degree of in vivo instability despite high stability in serum in vitro. Bone uptake is consistent with partial release of fluorine-18 as free fluoride, which preferentially localizes to mineralized tissue like bone and is cleared primarily via the kidneys.^38^

PET imaging of in vitro fluorine-18-labeled Jurkat cells showed rapid and high initial retention in the lungs and liver (Figure 6C). Over the 90 min imaging window, activity gradually redistributed from the lungs to the liver; notably, liver activity continued to rise throughout the scan, whereas in the tracer-only conditions, liver activity peaked and then declined, as reflected by the TACs (Figure 7A). Ex vivo biodistribution confirmed this pattern, with the highest uptake in lungs (66.74 ± 38.77% ID/g), liver (43.14 ± 22.39% ID/g), and spleen (29.1 ± 3.19% ID/g), the latter consistent with immune-cell retention in secondary lymphoid tissue. A similar lung first-pass retention, followed by redistribution, has been reported for other labeled Jurkat, CAR-T, and other cell models, for instance, iodine-124-labeled Jurkat cells in healthy NSG mice,^43^ zirconium-89-labeled CAR-Jurkat cells in tumor-bearing mice,^44^ labeled CAR-T cells,^45,46^ and zirconium-89-labeled murine myeloma cells,^47^ human mesenchymal stem cells,^48^ and dendritic cells.^49^ Mechanistically, prolonged pulmonary transit after intravenous infusion has been attributed to low-pressure pulmonary circulation and capillary narrowing during respiration, which can slow the passage of adoptively transferred T cells.^50^ Activated T cells may also exhibit longer pulmonary retention than naive counterparts.^51,52^ Furthermore, biodistribution is strongly dependent on the administration route; for example, indium-111-labeled CAR-T cells remained lung-retained after intravenous injection but remained localized to the peritoneal cavity after intraperitoneal injection, or near the injection site after subcutaneous administration.^45^

Despite successful initial cell labeling, substantial activity was detected in urine (21.33 ± 4.12% ID/g), comparable to the tracer-only group, consistent with partial loss of the radiolabel and renal clearance of released fluorine-18-labeled species. Gallbladder activity was also relatively high (14.60 ± 16.19% ID/g), whereas small intestine activity was comparatively low (5.28 ± 4.11% ID/g) relative to the tracer-only group. Taken together, these patterns suggest that the primary source of radiolabel loss from in vitro fluorine-18-labeled cells is decomplexation of the fluorine-18 radiolabel rather than release of intact **Al­[** ^**18**^ **F]­F-3**.

In the pretargeting experiments (Figure 6D–F), the distribution of activity closely resembled that of **Al­[** ^**18**^ **F]­F-3** alone (Figure 6B), as confirmed by both PET imaging and ex vivo biodistribution (Figures 7C and S22 and S23 and Table S7). A longer imaging window would be required to determine whether any liver-associated activity persists after systemic clearance of **Al­[** ^**18**^ **F]­F-3**, which could corroborate the in vivo Tz-TCO ligation. However, without metabolite analysis, it remains unclear whether the hepatic signal derives from the intact **Al­[** ^**18**^ **F]­F-3** tracer or radiolabeled metabolites, motivating future evaluation of Tz-based radiotracers with improved hydrophilicity and preferential renal clearance for this application.

Repeated measure analysis of the TACs indicated that the in vitro fluorine-18-labeled cells showed the most consistent differences to **Al­[** ^**18**^ **F]­F-3**, whereas the cell-receiving groups in the pretargeting experiments exhibited limited significant differences, primarily only in the kidneys where lower SUVs were observed for animals receiving the TCO-modified cells than those receiving only azide-modified cells or **Al­[** ^**18**^ **F]­F-3** alone (Table S6). While this might indicate the success of the IEDDA reaction in blood, the lack of differences in the TACs for organs in the hepatobiliary elimination route or in the heart does not support this. To quantify cumulative organ exposure, AUCs of the TACs were calculated over the 0–90 min imaging window (Figures 7B and S21B); the AUC analysis corroborated the qualitative PET and TAC observations. **Al­[** ^**18**^ **F]­F-3** exhibited dominant hepatobiliary exposure (highest AUCs in liver, small intestine, and gallbladder) with a moderate renal contribution. In vitro fluorine-18-labeled cells showed markedly elevated lung and liver AUCs, reflecting prolonged pulmonary retention followed by hepatic redistribution, and increased spleen AUC uniquely in this group, consistent with lymphoid sequestration. In contrast, pretargeting conditions displayed AUC profiles closely matching tracer-only control, suggesting that organ exposure was driven primarily by tracer pharmacokinetics rather than effective in vivo Tz-TCO ligation under the studied conditions. For a statistical comparison, the AUCs in cell-receiving groups in the pretargeted imaging experiment were tested against the **Al­[** ^**18**^ **F]­F-3** control using the Mann–Whitney *U* test, and no statistically significant differences between groups were observed.

Further, efficient in vivo pretargeting will also depend on the persistence of MGE-installed azide moieties on the cell surface, which is governed by membrane and glycoprotein turnover and can vary substantially across cell types. Kang and coauthors reported tracking of azide-modified A549 lung cancer cells for up to 3 days in vivo,^53^ while a recent study observed azide persistence on red blood cells for >42 days but loss from leukocytes within 3 days.^54^

Lastly, while the dual-bioorthogonal strategy offers modularity, it also introduces additional steps that may reduce the overall labeling efficiency. Alternative approaches could include either SPAAC-based radiolabeling of azide-modified cells or direct IEDDA-based radiolabeling following MGE of a dienophile-modified sugar. Direct SPAAC-based radiolabeling of azide-modified cells represents the most straightforward strategy. However, SPAAC-based ligations are significantly slower than IEDDA reactions, with second-order rate constants typically several orders of magnitude lower.^55^ While SPAAC has been widely used for fluorescence-based cell labeling, where probe concentrations can be relatively high and longer reaction times are acceptable, and has also proven useful for in vitro cell radiolabeling with longer-lived radionuclides,^8^ the relatively slow kinetics are less favorable for PET imaging applications using short-lived radionuclides such as fluorine-18, particularly for in vivo pretargeting. Further, as we demonstrate here, the lipophilicity and consequent nonspecific binding of DBCO reagents and their influence on the cell labeling efficiency need to be carefully assessed, which many studies overlook.

An alternative single-step approach could involve the MGE of dienophile-modified sugars,^56^ such as a TCO-modified sugar,^57^ followed by the direct ligation with a radiolabeled Tz. However, the metabolic acceptance of these sugars is highly dependent on their structure. In many cases, strained alkene reporters that are well tolerated display relatively low reactivity toward Tzs. Consequently, when developing new dienophile-modified sugars, the level of cell surface expression and their reactivity toward the bioorthogonal partner must be carefully evaluated. Furthermore, TCOs undergo trans–cis isomerization, leading to a drastic decrease in the TCO-Tz ligation yield. This process has been reported to occur in biological environments, including cell culture media.^58^ Whether this process is enhanced under MGE conditions has not been thoroughly investigated, but it may represent an additional limitation of the TCO-modified sugars. As a result, developing dienophile-modified sugars that combine efficient metabolic incorporation with high IEDDA reactivity remains a challenge. Other less established bioorthogonal reactions, such as the Tz-isonitrile ligation, have also been investigated in the context of MGE.^59^ However, their application to cell radiolabeling has, to the best of our knowledge, not yet been reported.

In contrast, azide-modified sugars such as Ac_4_MaNAz remain the most widely used and characterized metabolic reporters due to their small size, excellent metabolic tolerance, and robust incorporation across many cell types. The dual-click strategy presented here, therefore, represents a practical compromise, combining reliable azide-based MGE with rapid radiolabeling through fast Tz-TCO ligation.

## Conclusion

This work demonstrates that combining MGE with bioorthogonal chemistry provides a versatile, nongenetic platform for selective cell radiolabeling. The newly developed sulfo-DBCO-PEG_4_-TCO derivative enabled efficient sequential SPAAC-based and Tz-TCO ligation, and the Tz tracer **Al­[** ^**18**^ **F]­F-3** was produced with high RCC and RCP. This mild, cell-compatible labeling workflow maintained cell viability and provided radiolabel retention over time scales relevant for PET. Dynamic PET/CT imaging showed consistent early in vivo biodistribution of in vitro fluorine-18-labeled Jurkat cells, supporting the feasibility of this strategy for noninvasive monitoring of therapeutic cells after MGE. Although **Al­[** ^**18**^ **F]­F-3** showed some in vivo instability and predominantly hepatobiliary clearance—features that are not optimal for pretargeted imaging—and the dual-bioorthogonal strategy requires further optimization for upscaling to higher cell numbers and activities, these findings provide a useful foundation for the further development of Tz-based probes and bioorthogonal pretargeted strategies for cell tracking.

## Experimental Procedures

Detailed analytical methods, synthetic procedures, compound characterization data (NMR, HRMS, and LC–MS), synthesis of **Al** ^**nat**^ **F-3** reference and analysis of its decomplexation under HPLC conditions, radiosynthesis, and in vitro evaluation of **Al­[** ^**18**^ **F]­F-3**, protocols for flow cytometry and in vitro cell radiolabeling for exploratory experiments, and in vivo evaluation experimental details, additional flow cytometry and in vitro cell radiolabeling graphs, grayscale standalone PET images, additional biodistribution graphs, and values are provided in the Supporting Information. No unexpected or unusually high safety hazards were encountered using the methods described, and all handling of radioactive materials was carried out in laboratories designated for radioactive work with the activity levels used with appropriate shielding (e.g., in lead-shielded hot cells and fume hoods) and donning appropriate personal protective equipment and dosimeters. All animal experiments were conducted under a project license approved by the National Board of Animal Experimentation in Finland (license number ESAVI/17892/2025) and in accordance with the respective national and European Union legislation.

## Supplementary Material













## Notes

Detailed materials and experimental protocols (synthesis of **1**, **3**, and **Al** ^**nat**^ **F-3**, in vitro SPAAC-based and TCO-Tz ligations, radiosynthesis and in vitro evaluation of **Al­[** ^**18**^ **F]­F-3**, flow cytometry, cell radiolabeling, and animal studies); NMR and HRMS spectra, LC–MS analysis, additional HPLC analyses, radio-TLC examples, additional flow cytometry and cell radiolabeling graphs, grayscale PET images, additional TACs and AUCs, summaries of statistical analysis, additional biodistribution graphs, and tabulated values (PDF)
Dynamic PET/CT movie of **Al­[** ^**18**^ **F]­F-3** (GIF)
Dynamic PET/CT movie of in vitro-fluorine-18-labeled cells (GIF)
Dynamic PET/CT movie of TCO-modified cells followed by **Al­[** ^**18**^ **F]­F-3** injection after 30 min (GIF)
Dynamic PET/CT movie of TCO-modified cells followed by **Al­[** ^**18**^ **F]­F-3** injection after 120 min (GIF)
Dynamic PET/CT movie of azide-modified cells followed by **Al­[** ^**18**^ **F]­F-3** injection after 120 min (GIF)

## Abbreviations

[^18^F]­F^–^: no-carrier-added [^18^F]­fluoride
[^18^F]­FHBG: 9-[4-[^18^F]­fluoro-3-(hydroxymethyl)­butyl]­guanine
[^18^F]­TFB: ^18^F-tetrafluoroborate
[^18^F]­DCFPyL: Piflufolastat F-18
Ac_4_ManNAz: tetra-acetylated *N*-azidoacetylmannosamine
ACT: adoptive cell therapy
Al­[^18^F]­F: aluminum [^18^F]­fluoride
*A*_m_: molar activity
AUC: area under the curve
BioD: biodistribution
CAR-T: chimeric antigen receptor T cells
CuAAC: copper-catalyzed azide–alkyne cycloaddition
DBCO: dibenzocyclooctene
DMSO: dimethyl sulfoxide
DPBS: Dulbecco’s phosphate-buffered saline
EtOH: ethanol
FBS: fetal bovine serum
HBSS: Hanks’ balanced salt solution
HPLC: high performance liquid chromatography
HRMS: high resolution mass spectrometry
HSV1-tk: herpes simplex virus type 1 thymidine kinase
hNIS: human sodium iodide symporter
IEDDA: inverse electron-demand Diels–Alder
iv: intravenous
ID/g: injected dose per gram of tissue
LC–MS: liquid chromatography–mass spectrometry
logD: distribution coefficient
MBq: megabecquerel
MFI: median fluorescence intensity
MGE: metabolic glycoengineering
MIP: maximum intensity projection
NK: natural killer
NMR: nuclear magnetic resonance
NODA: 1,4,7-triazacyclononane-1,4-diacetic acid
NOTA: 1,4,7-triazacyclononane-1,4,7-triacetic acid
OI: optical imaging
OVA-CTLs: ovalbumin-specific cytotoxic T lymphocytes
PBS: phosphate-buffered saline
PBS-T: phosphate-buffered saline containing Tween 20
PET: positron emission tomography
PSMA: prostate-specific membrane antigen
PS: penicillin–streptomycin
QC: quality control
RCC: radiochemical conversion
RCP: radiochemical purity
radio-HPLC: radio-high performance liquid chromatography
radio-TLC: radio-thin layer chromatography
RESCA: REstrained Complexing Agent
ROI: region of interest
SPE: solid-phase extraction
SPAAC: strain-promoted alkyne–azide cycloaddition
SPECT: single-photon emission computed tomography
SUV: standardized uptake value
TAC: time–activity curve
TCO: *trans*-cyclooctene
