# Supplementary material for: Metabolic Glycoengineering Enables Fluorine-18 Radiolabeling of T Lymphocytes via Dual-Bioorthogonal Chemistry
Source: Bioconjug Chem. 2026 Apr 24;37(5):981–94. doi: 10.1021/acs.bioconjchem.6c00052 (PMC13195574; doi:10.1021/acs.bioconjchem.6c00052)
Supplement: Supplementary file 1 [file bc6c00052_si_001.pdf]

Supporting Information

Metabolic glycoengineering enables fluorine-18 radiolabeling of T lymphocytes via dual-bioorthogonal chemistry

Anisa Biti[†], Alessia Centanni[†], Surachet Imlimthan[†], Heidi Harjunpää[‡], Arina Sukhova[†], Susanne K. Wiedmer[†], Susanna Fagerholm[‡] & Mirkka Sarparanta[†]*

[†]Department of Chemistry, Faculty of Science, University of Helsinki, 00560 Helsinki, Finland

[‡]Department of Molecular and Integrative Biosciences, Faculty of Biological and Environmental Sciences, University of Helsinki, 00790 Helsinki, Finland

*Corresponding author: Mirkka Sarparanta (mirkka.sarparanta@helsinki.fi)

Table of Contents

Additional abbreviations 4

Experimental 5

Materials and reagents 5

Analysis and purification 5

Liquid chromatography methods 6

HRMS analysis 7

Synthesis and NMR characterization of the bioorthogonal partners (Scheme 1) 8

Figure S1. ^{1}H NMR spectra of final compound **1** in MeOD. 9

Figure S2. ^{13}C NMR of final compound **1** in MeOD. 9

Figure S3. COSY NMR of final compound **1** in MeOD. 10

Figure S4. HSQC NMR of final compound **1** in MeOD. 10

Figure S5. HMBC NMR of final compound **1** in MeOD. 11

Figure S6. ^{1}H NMR spectra of final compound **3** in DMSO-*d*$_6$. 13

.... 13

Figure S7. ^{13}C NMR of final compound **3 in** DMSO-*d*$_6$. 13

Figure S8. HRMS spectra of final compound **1**. 14

Figure S9. HRMS spectra of final compound **3**. 14

Table S1. HRMS characterization data for final compounds **1** and **3**. 15

Investigation of the non-radioactive complex Al^{nat}F-3 15

Scheme S1. Synthesis of **Al^{19}F-3**. 15

Results: HPLC and ^{19}F NMR analysis 15

Figure S10. Analysis of non-radioactive complex **Al^{nat}F-3**. 17

Radiochemistry 17

Reagents and materials 17

Radiosynthesis of Al[^{18}F]F-3 (Scheme 2) 18

Figure S11. Radio-TLC example of a reaction mixture after 30 min: starting activity of 746 MBq and total reaction volume of 640 μL. 19

Figure S12. Example of a radio-TLC analysis of a QC sample of **Al[^{18}F]F-3** after Alox N cartridge purification. 19

In vitro evaluation of Al[^{18}F]F-3 20

Radiolabel stability (Figure 2 and Figure S13) 20

Figure S13. Additional stability graphs. 20

Lipophilicity 21

Molar activity 21

In vitro bioorthogonal ligations (Figure 2 and Figures S14–S16) 22

Scheme S2. **A**. SPAAC-based ligation between Ac$_4$ManNAz and **1** (only one possible regioisomer shown). **B**. Tz-TCO ligation between **1** and **3**/**Al[^{18}F]F-3**. 22

Figure S14. HPLC analysis of **1** (**A**) and SPAAC-based ligation reaction mixture after 30 min **(B)** at 254 nm. 23

Figure S15. LC-MS analysis of the Tz-TCO ligation reaction mixture between **1** and **3** 23

Figure S16. Extracted MS spectra of the LC-MS analysis of the Tz-TCO ligation mixture between **1** and **3**. .24

Cell culturing 25

Reagents and materials 25

Flow cytometry 25

Protocol 1: evaluation of DBCO-PEG$_2$-TCO......25
Protocol 2: evaluation of compound 1......26
Flow cytometry results: evaluation of DBCO-PEG$_2$-TCO......27
Figure S17. Flow cytometry analysis of two-step cell-surface labeling using 10 μM DBCO-PEG$_2$-TCO (**A**) or 20 μM DBCO-PEG$_2$-TCO (**B**). 28
Table S2. Quantitative flow cytometry analysis following two-step labeling using **Protocol 1** expressed as percentage of Cy3-positive cells and median fluorescence intensity (MFI) for samples shown in **Figure 3A**..29
Table S3. Quantitative flow cytometry analysis following two-step labeling using **Protocol 2** expressed as percentage of Cy3-positive cells and median fluorescence intensity (MFI) for samples shown in **Figure 3B**..29

***In vitro cell radiolabeling*......29**

Protocol 1: initial experiments......30
Figure S18. Schematic overview of protocol 1 for initial MGE-based cell radiolabeling experiments. 31
Table S4. Summary of experimental conditions evaluated for cell radiolabeling under **Protocol 1**. 32

Protocol 2: optimized conditions......32
Table S5. Summary of experimental conditions evaluated for cell radiolabeling under **Protocol 2**. The incubation with **Al[^{18}F]F-3** was done at 37 ºC for 30 min. 33

Cell labeling results......34
Figure S19. Cell-associated activity (%) of control and metabolically glycoengineered Jurkat cells following SPAAC-based and Tz-TCO ligations under different conditions. 37

***Animal studies* 37**

Cell labeling for animal studies 37

In vivo evaluation......38

Dynamic PET/CT imaging and ex vivo biodistribution......39
Figure S20. Representative summed PET images in grayscale 40
Figure S21. Additional TACs and AUCs. 41
Table S6. Statistical comparison of time–activity curves (TACs) between control (**Al[^{18}F]F-3**) and cell-based conditions using the Mann–Whitney test. 42
Figure S22. Complete ex vivo biodistribution 43
Table S7. Ex vivo biodistribution values after a 90 min PET/CT dynamic scan. 44
Figure S23. Ex vivo biodistribution in selected organs per condition. 45

***Statistical methods* 46**

Additional abbreviations

2D: Two-dimensional

7-AAD: 7-aminoactinomycin D

AcOH: acetic acid

ACN: acetonitrile

$AlCl_3 \cdot 6H_2O$: aluminum chloride hexahydrate

DMF: dimethylformamide

DMSO: dimethyl sulfoxide

DPBS: Dulbecco's phosphate buffer saline

MeOD: deuterated methanol

MeOH: methanol

NaOAc: sodium acetate

FACS: fluorescence-activated cell sorting

HUS: Helsinki University Hospital

SPE: solid-phase extraction

TFA: trifluoroacetic acid

Experimental

Materials and reagents

Tz-NHS ester, sulfo-DBCO-PEG_4-amine, DBCO-PEG_2-TCO, and TCO-NHS ester (BroadPharm, San Diego, CA), sulfo-Cy3-Tz and Ac_4ManNaz (Lumiprobe, Westminster, MD, USA), acetonitrile (ACN), methanol (MeOH), sodium acetate (99.99%), N,N-Diisopropylethylamine (DIPEA), anhydrous dimethylformamide (DMF), anhydrous dichloromethane (CH_2Cl_2), anhydrous dimethylsulfoxide (DMSO), anhydrous chloroform ($CHCl_3$), aluminum chloride hexahydrate ($AlCl_3 \cdot 6H_2O$, 99.99%) from Sigma Aldrich (St. Louis, MO, USA), tert-Butyl (trans-4-(aminomethyl)cyclohexyl)carbamate (BLDPharm, Shanghai, China), and (+)-RESCA-TFP (CheMatech, Dijon, France) were used without purification. Other commonly used solvents, such as EtOH and acids such as trifluoroacetic acid (TFA), glacial acetic acid (AcOH), and formic acid (FA), were purchased from Thermo Fisher Scientific.

Analysis and purification

TLC was done using aluminum-backed silica plates with F254 indicator (Sigma Aldrich or Macherey-Nagel), visualized under a UV lamp and/or staining with a potassium permanganate ($KMnO_4$) or ninhydrin staining solution. Compounds were purified by manual silica flash chromatography, reversed-phase flash column chromatography using RediSep Gold® C18 Reversed-Phase columns and Teledyne Isco Combiflash Ez Prep (Teledyne Technologies, Thousand Oaks, CA, USA) or by preparative HPLC (method B). An Alpha 1–4 LSC lyophilizer (Martin Christ, Osterode am Harz, Germany) was used to lyophilize samples after purification.

Liquid chromatography methods

HPLC analysis (method A) and purification (method B) was performed using a Shimadzu Prominence (Tokyo, Japan) HPLC system with a DGU-20A degasser, an LC-20AD UPLC LC unit, a SIL-20A HT autosampler, a CTO-20AC column oven, a CBM-20A communications bus module, a SPD-M20A diode array detector (DAD) or ultraviolet (UV) and a Scionix Holland scintillation detector with a 51 BP 51/2 NaI(Tl) crystal for radiodetection. Data was processed with LabSolutions software (version 5.87) from Shimadzu.

Method A (HPLC and radio-HPLC analysis)

Column: Hichrom Alltima or Grace Alltima 5 μm 100 Å, 250×4.6 mm; Eluent A: 0.1% TFA in mQ; Eluent B: 0.1% TFA in ACN; Gradient: 0–5 min 5% B, 5–17.5 min 5–95% B, 17.5–22.5 min 95% B, 22.2–25 min 95–5% B, 25–30 min 5% B.

Method B (preparative HPLC)

Column: Atlantis T3 OBD Prep, 100Å, 10 μm, 250 mm×10 mm, flow rate 3 mL/min; Eluent A: 0.1% TFA in mQ; Eluent B: 0.1% TFA in ACN; Gradient: 0–10 min 5–30% B, 10–15 min 30% B, 15–20 min 30–50% B, 20–27 min 50% B, 27-32 min 50–95% B, 32–37 min 95% B, 37–40 min 95–5% B, 40–45 min 5% B.

LC-MS analysis was performed using Agilent Technologies 1260 Infinity HPLC-DAD system with Agilent Technologies 6120 Quadruple LC/MS detector (Agilent Technologies, Santa Clara, CA, USA); Ionization HESI+, scan range 100-2000 m/z, and an Agilent InfinityLab Poroshell

120EC-C18 (4 mm, 4.6Å~100 mm) column. Data was processed with Agilent OpenLAB CDS (version 2.2).

Method C (LC-MS method)

Column: Agilent Poroshell 120EC-C18 (4 mm, 4.6Å~100 mm), flow rate 1 mL/min; Eluent A: 0.1% FA in mQ; Eluent B: 0.1% FA in ACN; Gradient: 0–5 min 5% B, 5–17 min 95% B, 17–20 min 95% B, 20–22 min 5% B, 22–25 min 5% B.

HRMS analysis

HRMS spectra of the final compounds were recorded on a Q Exactive HF Orbitrap mass spectrometer (Exactive series, Thermo Fisher Scientific, Bremen, Germany) with HESI ion.

Samples were prepared by dilution of DMSO stock solutions in methanol to obtain concentrations 1 µg/mL for futher infusion with a syringe pump (CHEMYX Fusion 100T) and 500 µL Hamilton syringe at flow of 5 µL/min. Orbitrap MS with the heated electrospray ion source was operated in negative polarity ion mode. Spray voltage was set to -3 kV and capillary temperature to 320 °C. Nitrogen gas was used as a sheath gas with a flow rate 10 arbitrary units. S-lens radio frequency level was set to 100. Resolution was set to 240 000, automatic gain control to 1×10^6, maximum injection time to 100 ms. External calibration was done using Pierce ESI Negative Ion Calibration Solution (Thermo Fisher Scientific Inc., Rockford, USA). Data was processed with Thermo Xcalibur Qual Browser (version 4.5.474.0). The error between the measured and theoretical exact mass was calculated and reported in parts per million (ppm) as shown in **Table S1**.

Synthesis and NMR characterization of the bioorthogonal partners (Scheme 1)

^{1}H NMR, ^{13}C NMR, and 2D NMR spectra were acquired using a 400 MHz Bruker Avance NEO with autosampler and a 500 MHz Bruker Avance NEO NMR spectrometer with a 5 mm probe and processed with MestreNova 14.2.2 software, using non-deuterated residual solvent peaks as references.

Sulfo-DBCO-PEG$_4$-TCO (1) was synthesized in two steps starting from commercially available reagents. To a solution of sulfo-DBCO-PEG$_4$-amine (1.2 eq., 5 mg, 7.4 μmol) and DIPEA (3 eq., 3.9 μL, 22.2 μmol) in 0.3 mL anhydrous DMSO, was added TCO-NHS ester (1 eq., 1.7 mg, 6.2 μmol), dissolved in anhydrous DMF (0.2 mL); the reaction was stirred at rt for 2 h and monitored by HPLC (method A). The mixture was loaded onto a Sep-Pak C18 Plus Short cartridge (Waters, Milford, MA, USA), followed by washing with 2–4 mL mQ and elution with 25–50% ACN in mQ. Fractions were combined and lyophilized to afford compound **1** as a white solid, 3.1 mg in 50.2% yield.

^{1}H NMR (500 MHz, MeOD) δ 7.70 – 7.65 (m, 1H), 7.64 – 7.56 (m, 1H), 7.51 – 7.42 (m, 3H), 7.41 – 7.31 (m, 2H), 7.30 – 7.25 (m, 1H), 5.65 – 5.55 (m, 1H), 5.52 – 5.43 (m, 1H), 5.17 (dd, J = 14.0, 3.1 Hz, 1H), 4.31 (s, 1H), 3.75 – 3.46 (m, 20H), 3.30 – 3.16 (m, 5H), 2.68 – 2.59 (m, 2H), 2.45 – 2.26 (m, 5H), 2.08 – 1.87 (m, 5H), 1.78 – 1.53 (m, 3H);

^{13}C NMR (126 MHz, MeOD) δ 173.7, 173.3, 168.8, 158.7, 152.7, 149.4, 136.1, 133.7, 130.8, 129.6, 129.2, 128.0, 71.5, 71.4, 71.3, 71.2, 68.1, 66.1, 49.6, 49.5, 49.4, 49.3, 49.1, 49.0, 48.8, 48.6, 48.4, 42.2, 41.6, 39.6, 37.6, 35.1, 33.5, 32.1.

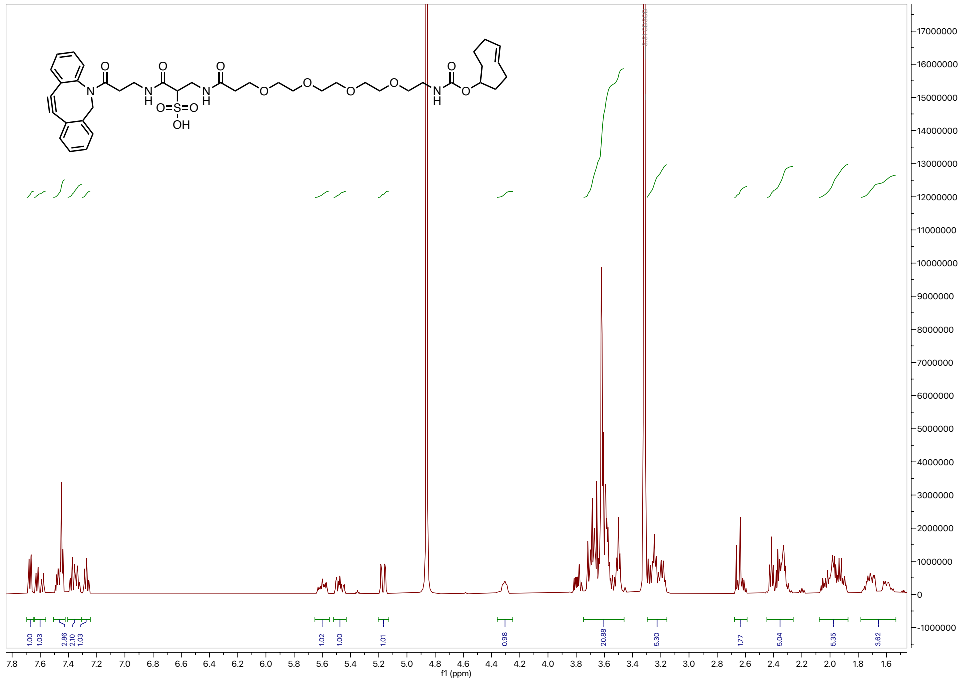

Figure S1. ^{1}H NMR spectra of final compound **1** in MeOD.

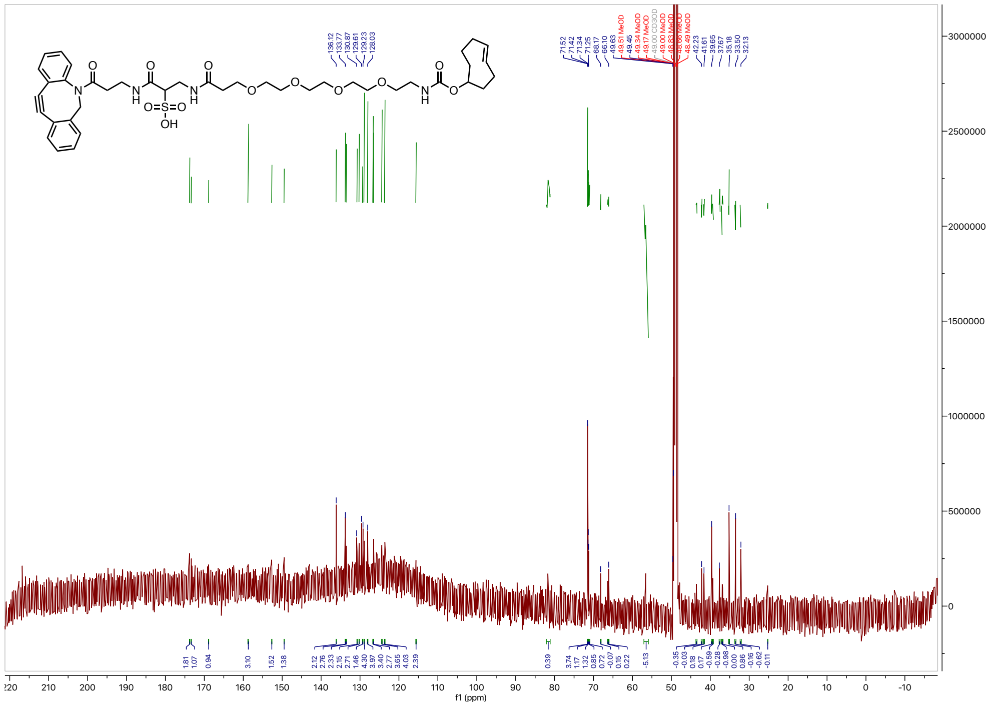

Figure S2. ^{13}C NMR of final compound **1** in MeOD.

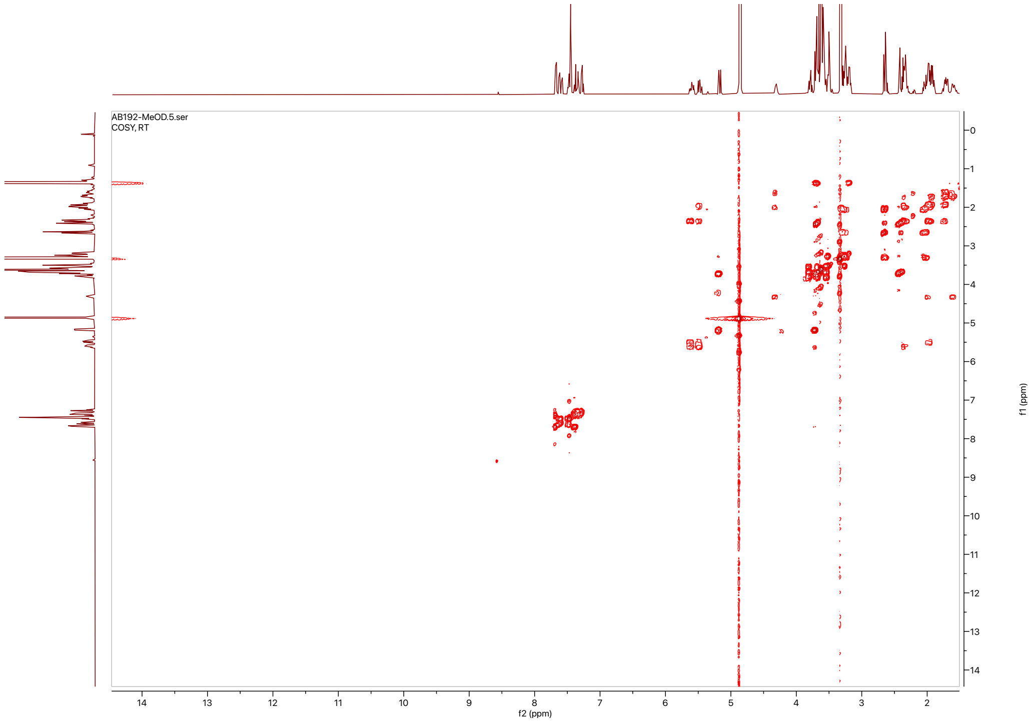

Figure S3. COSY NMR of final compound **1** in MeOD.

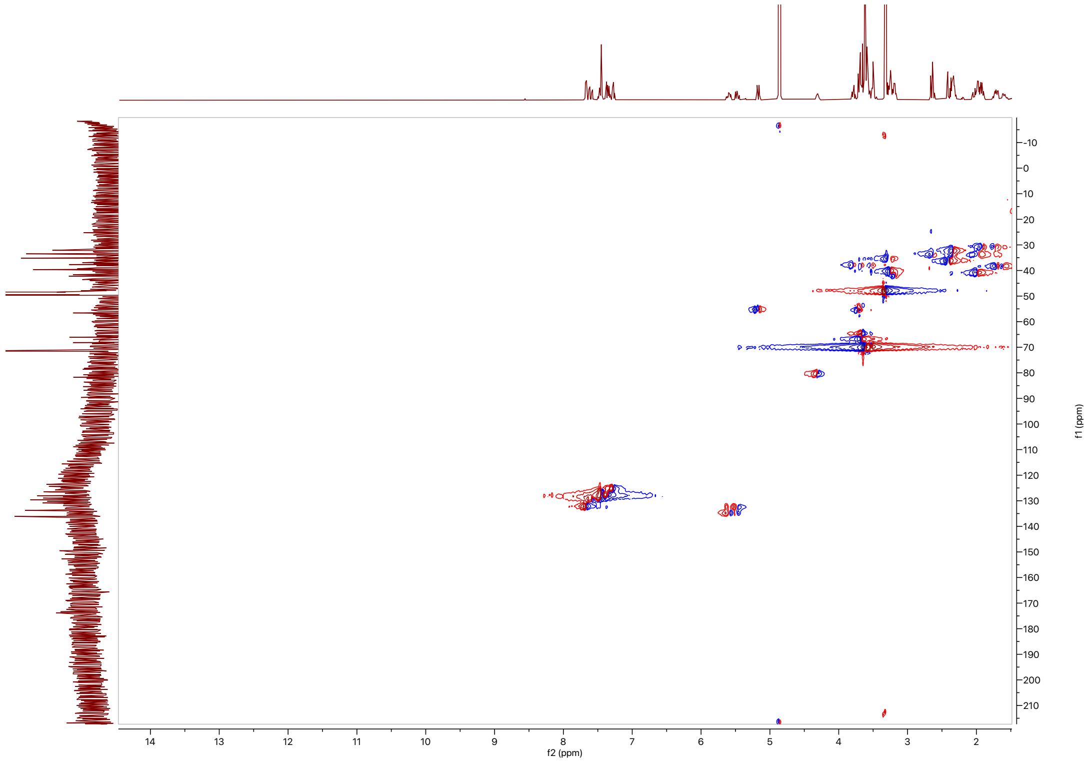

Figure S4. HSQC NMR of final compound **1** in MeOD.

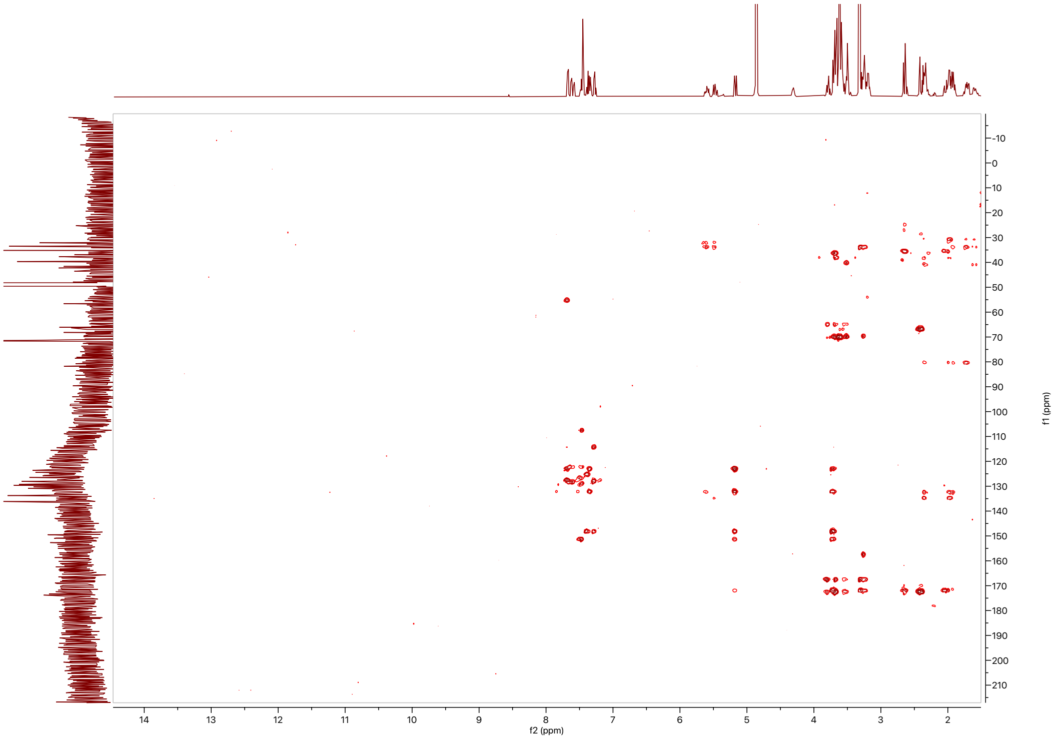

Figure S5. HMBC NMR of final compound **1** in MeOD.

(+)-RESCA-Tz (**3**) was synthesized in three steps starting from commercially available reagents. To a solution of tetrazine-NHS ester (1 eq., 25 mg, 79.8 μmol) in 1.5 mL anhydrous CH_2Cl_2 in a 4-mL amber glass vial, under argon (Ar) atmosphere, DIPEA was added, followed by the addition of tert-Butyl (trans-4-(aminomethyl)cyclohexyl)carbamate (1.1 eq., 20 mg, 87.8 μmol) as a solid in one portion. The reaction was stirred at rt for 2 h while being monitored by HPLC (method A). Then, after, it was evaporated to dryness, redissolved in anhydrous DMSO (1 mL), and purified using RediSep Gold® C18 Reversed-Phase column. The fractions were lyophilized to obtain 16.5 mg of compound **2** as a pink solid in 48.5% yield.

^{1}H NMR (400 MHz, DMSO) δ 10.57 (s, 1H), 8.49 – 8.39 (m, 2H), 8.11 (t, J = 5.7 Hz, 1H), 7.61 – 7.50 (m, 2H), 6.65 (d, J = 8.1 Hz, 1H), 3.56 (s, 2H), 3.32 (s, 2H), 3.18 – 3.05 (m, 1H), 2.91 (t, J = 6.2 Hz, 2H), 1.82 – 1.61 (m, 4H), 1.36 (s, 9H), 1.14 – 1.02 (m, 2H), 0.98 – 0.82 (m, 2H);

^{13}C NMR (101 MHz, DMSO) δ 182.2, 178.8, 174.9, 167.5, 164.2, 151.4, 139.5, 137.1, 86.7, 58.69, 54.1, 51.8, 49.6, 49.6, 49.4, 49.4, 49.2, 49.2, 49.0, 49.0, 48.7, 48.5, 48.3, 46.2, 41.6, 38.6, 37.7, 34.6.

Compound **2** (8.2 mg, 19.86 µmol) was dissolved in a 50:50 (v/v) mixture of CH_2Cl_2:TFA in a 4-mL amber glass vial, and the mixture was stirred at rt for 1 h. A sample of the reaction mixture was analyzed using HPLC. The solvent was evaporated under vacuum, and the crude compound was washed with 3–5 mL of $CHCl_3$ multiple times until it began to solidify. The TFA salt was used directly for the next step, without further analysis or purification; it was dissolved in anhydrous DMF (1 mL) under Ar atmosphere, and DIPEA (10 eq., 33.4 µL, 192 µmol) was added. In the meantime, (+)-RESCA-TFP (1.5 eq., 28.8 µmol) was weighed and added in one portion as a solid. The reaction was stirred at rt for 2 h and monitored by HPLC (method A). DMF was partially removed under vacuum, and the mixture was purified using preparative HPLC (method B) to afford compound **1** as a pink solid, 10.4 mg in 72.7 % yield.

^{1}H NMR (400 MHz, DMSO) δ 10.57 (s, 1H), 8.49 – 8.39 (m, 2H), 8.13 (t, J = 5.7 Hz, 1H), 7.90 (d, J = 7.8 Hz, 1H), 7.59 – 7.51 (m, 2H), 7.37 (d, J = 7.8 Hz, 2H), 7.17 (d, J = 7.8 Hz, 2H), 4.06 (d, J = 12.7 Hz, 2H), 3.73 (d, J = 12.7 Hz, 2H), 3.47 – 3.28 (m, 14H), 2.91 (h, J = 11.9 Hz, 5H), 2.10 – 1.91 (m, 3H), 1.83 – 1.59 (m, 6H), 1.29 – 1.04 (m, 7H), 1.02 – 0.81 (m, 3H);

^{13}C NMR (126 MHz, DMSO) δ 169.42, 168.83, 165.48, 158.38, 158.12, 157.86, 157.61, 141.96, 130.15, 130.02, 129.27, 127.68, 120.42, 118.05, 115.68, 113.31, 47.95, 44.65, 42.32, 42.05, 40.43, 40.11, 40.02, 39.95, 39.85, 39.69, 39.52, 39.35, 39.19, 39.02, 36.81, 31.93, 29.13, 23.94, 23.70.

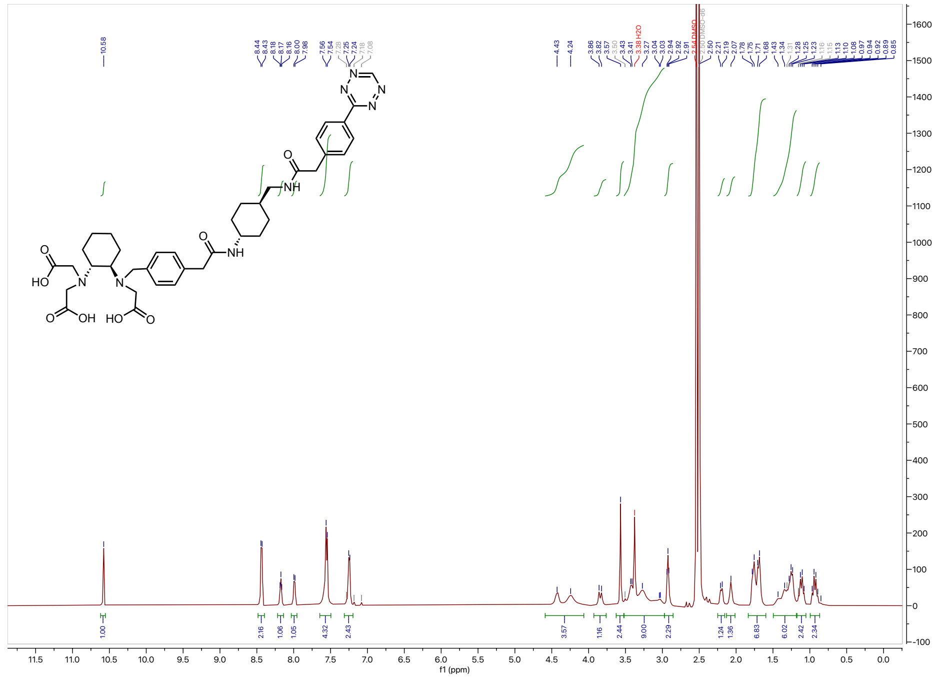

Figure S6. ^{1}H NMR spectra of final compound **3** in DMSO-d_6.

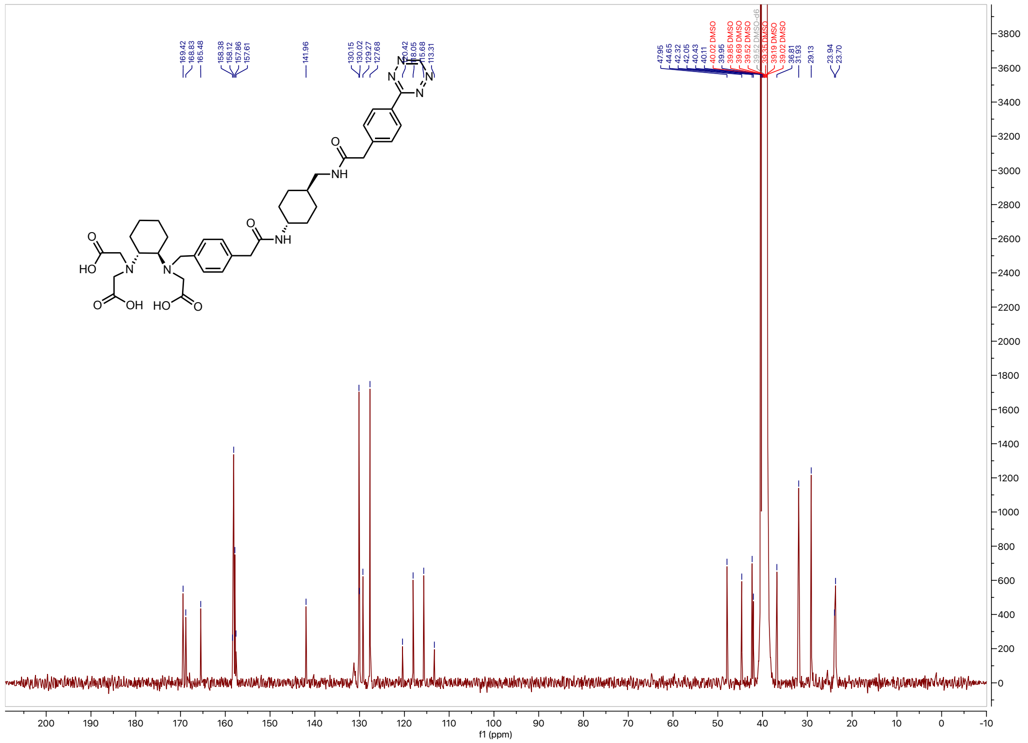

Figure S7. ^{13}C NMR of final compound **3 in** DMSO-d_6.

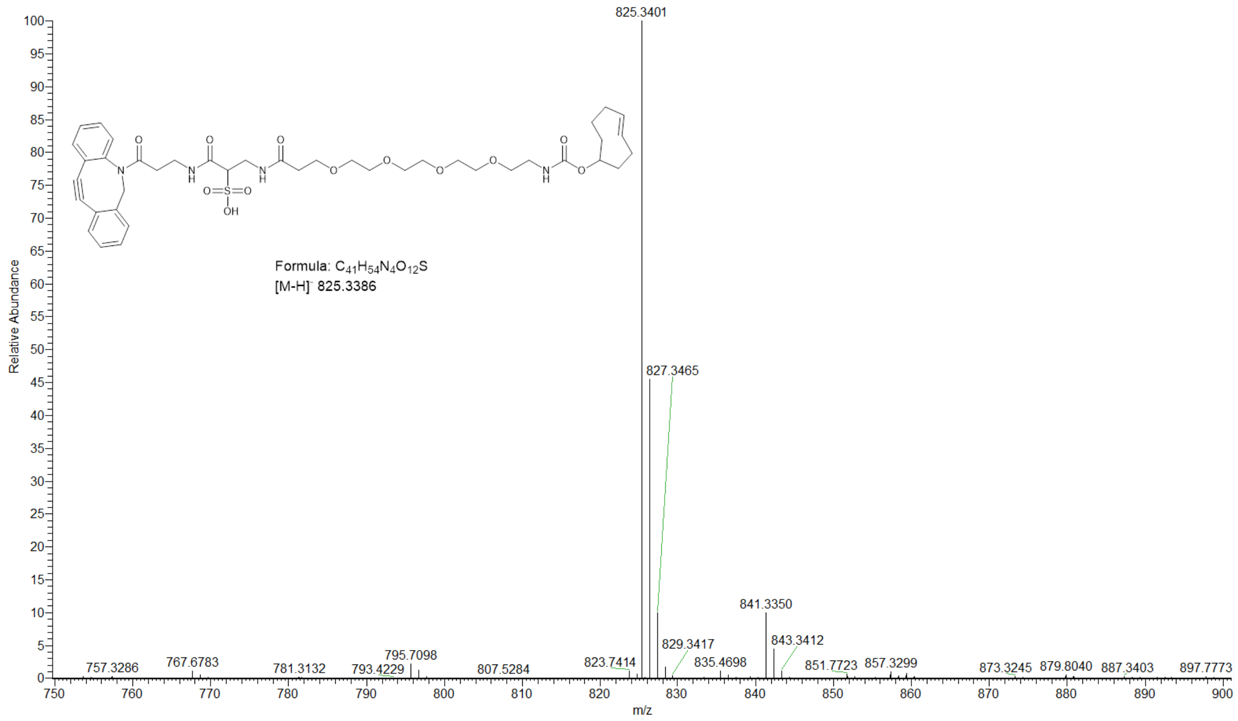

Figure S8. HRMS spectra of final compound **1**.

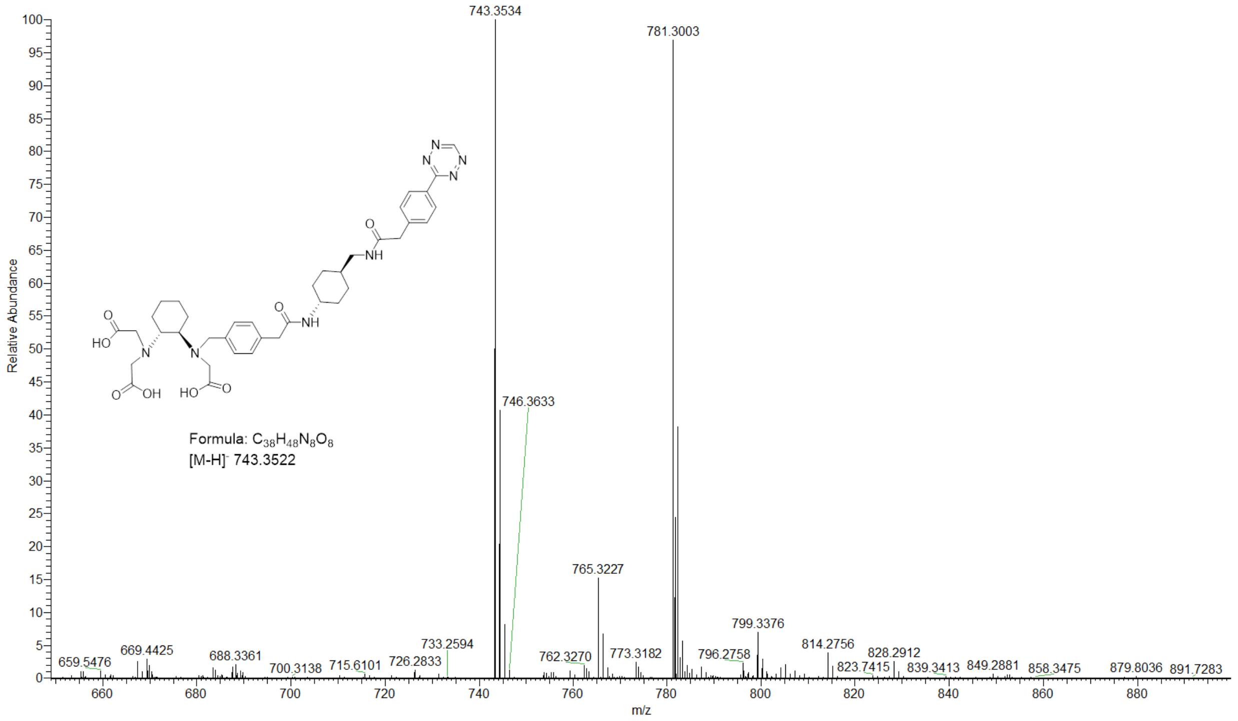

Figure S9. HRMS spectra of final compound **3**.

Table S1. HRMS characterization data for final compounds **1** and **3**.

Compound	Formula	Observed ions	Measured m/z	mass error (ppm)
**1**	$C_{41}H_{54}N_4O_{12}S$	$[M-H]^-$	825.3401	-1.82
**3**	$C_{38}H_{48}N_8O_8$	$[M-H]^-$	743.3534	-1.61

Investigation of the non-radioactive complex Al^{nat}F-3

A mixture of $AlCl_3$ (5 eq., 44.3 μmol, 443 uL of a 100 mM solution of $AlCl_3 \cdot 6H_2O$ in 0.1 M NaOAc pH 4.5) and Na^{nat}F (5 eq., 443 μL, of a 100 mM solution in 0.1 M NaOAc pH 4.5) was stirred at rt for 15 min. The mixture was added to **3** (1 eq., 6.6 mg, 8.9 μmol) and 886 μL of absolute EtOH was added to the mixture to achieve a final 1:1 (v/v) aqueous to organic solvent ratio. The reaction mixture was stirred at rt for 2 h and monitored by HPLC (method A).

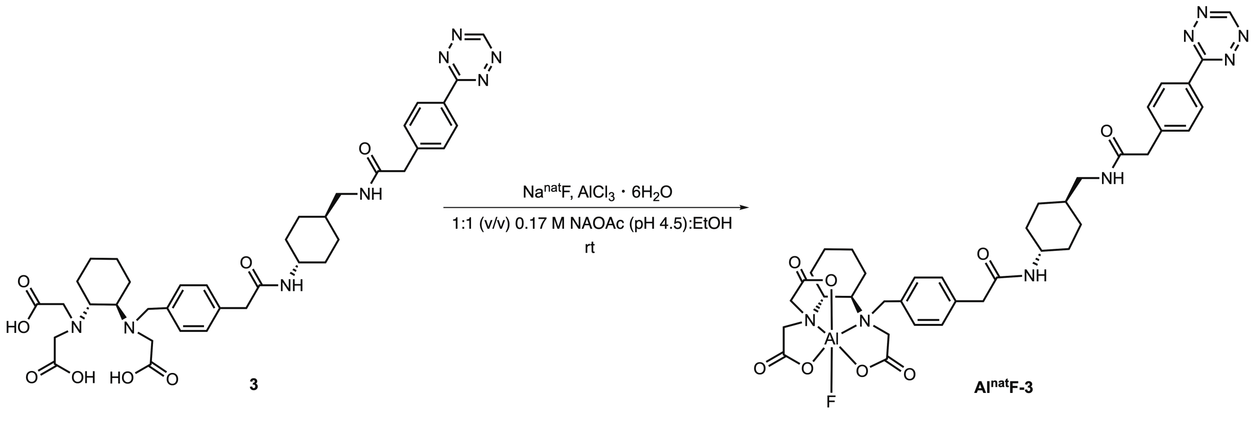

Scheme S1. Synthesis of **Al^{nat}F-3**.

Results: HPLC and ^{19}F NMR analysis

HPLC analysis (**Figure S10A**) and ^{19}F NMR analysis (**Figure S10B**) of the crude reaction mixture confirmed formation of an Al^{nat}F complex. However, following preparative HPLC purification (method B), analysis of the purified and lyophilized fractions indicated the presence of both precursor **3** and **Al^{nat}F-3** at approximately the same ratio as in the crude mixture (~56.5% and

~46.5%, respectively). This might be attributed to the partial decomplexation of the $Al^{nat}F$-3 under the acidic mobile phase conditions used for HPLC (pH 2–2.5). Metal-ligand complexes can dissociate rapidly at low pH,[37] and similar behavior has been reported for Al[^{18}F]F complexes with acyclic chelators.[38] No further attempts were made to isolate **$Al^{nat}F$-3**, as these acidic conditions are not representative of the in vitro and in vivo experiments in this study. Notably, the stability of the corresponding radioactive complex was supported by radio-TLC; therefore, we did not pursue further optimization of the HPLC conditions.

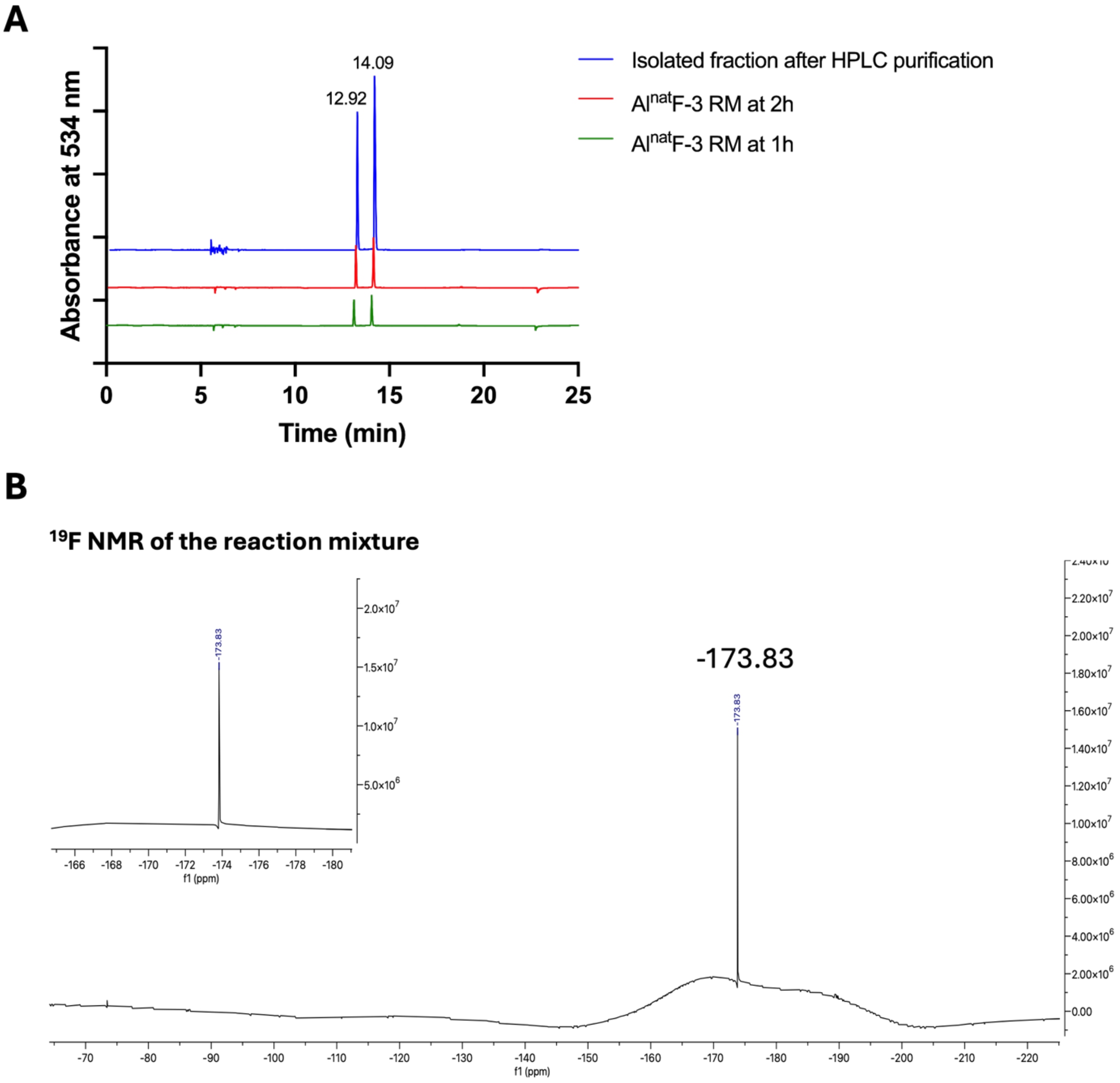

Figure S10. Analysis of non-radioactive complex **Al^{nat}F-3**. **A**. HPLC analysis of the **Al^{nat}F-3** synthesis monitored by HPLC at 534 nm (precursor **3** at 14.2 min and **Al^{nat}F-3** at 13.2 min). Chromatograms of the reaction mixture after 1 h (green) and 2 h (red) are shown alongside the isolated product obtained after HPLC purification (blue). B. ^{19}F NMR spectrum of the **Al^{nat}F-3** reaction mixture showing a single characteristic peak at δ −173.83 ppm.

Radiochemistry

Reagents and materials

No-carrier-added [^{18}F]fluoride was produced via the $^{18}O(p,n)^{18}F$ reaction by irradiating oxygen-18-enriched water ([^{18}O]H_2O) with 10-MeV protons on an IBA Cyclone 10/5 medical cyclotron (Louvain-la-Neuve, Belgium) in-house or with 18-MeV protons on an IBA Cyclone Kiube at the Cyclotron Unit, Helsinki University Hospital. [^{18}O]H_2O was purchased from various vendors, including Rotem Industries (Arava, Israel; 98% isotopic enrichment), Campro Scientific (Berlin, Germany; 97% enrichment), and Taiyo Nippon Sanso (Tokyo, Japan; 98% enrichment).

Radioactivity was measured using a dose calibrator (Capintec Radioisotope Calibrator CRC-721, Ramsey, NJ, USA). The radiolabeled compounds were analyzed using iTLC-SG from Agilent (Santa Clara, CA, USA, product no. SGI0001) with 0.5% AcOH in MeOH (v/v) as an eluent. After air-drying, the iTLC-SG sheet was exposed to a digital imaging plate for photostimulated luminescence (PSL) detection (Fuji BAS-TR2025, 20×25 cm) for an appropriate period, depending on the activity. The imaging plate was scanned on a Fujifilm Fluorescent Image

Analyzer (FLA-5100 V.1, Fuji Film Photo, Tokyo, Japan). The radio-TLCs were analyzed using AIDA image analysis software version 5.0 SP 3 (Elysia-Raytest GmbH, Straubenhardt, Germany). All buffers used for radiolabeling were treated with Chelex (100 sodium form, Sigma-Aldrich, St. Louis, MO, USA) and filtered with 0.22 μm filters. Sep-Pak Alumina N Plus Light Cartridge was purchased from Waters (Milford, MA, USA), while Chromafix Alox N was purchased from Macherey-Nagel (Düren, Germany).

Radiosynthesis of Al[^{18}F]F-3 (Scheme 2)

60 nmol of $AlCl_3$ (30 μL of a 2 mM solution of $AlCl_3 \cdot 6H_2O$, 99.99%) in 0.17 M NaOAc (pH 4.5) and 0.5 μL of AcOH were incubated with [^{18}F]F^- (50–300 μL of [^{18}O]H_2O containing ca. 123–1243 MBq) at rt for 10 min. Then, 120 nmol of precursor **2** (in 10 μL DMSO) dissolved in 0.17 M NaOAc (pH 4.5): EtOH was added to achieve a final 1:1 (v/v) aqueous to organic solvent ratio (final volume 370–940 μL). The reactions were incubated at rt for 30 min. RCC was determined using iTLC-SG and 0.5% AcOH in MeOH as an eluent. **Al[^{18}F]F-3** was purified on a Chromafix Alox N or Sep-Pak Alumina N Plus Light Cartridge without preconditioning and eluted with 1:1 (v/v) 1×PBS (pH 7.4): EtOH. The purified product was analyzed by radio-iTLC and radio-HPLC. **Figures S11** and **S12** show selected radio-TLC examples.

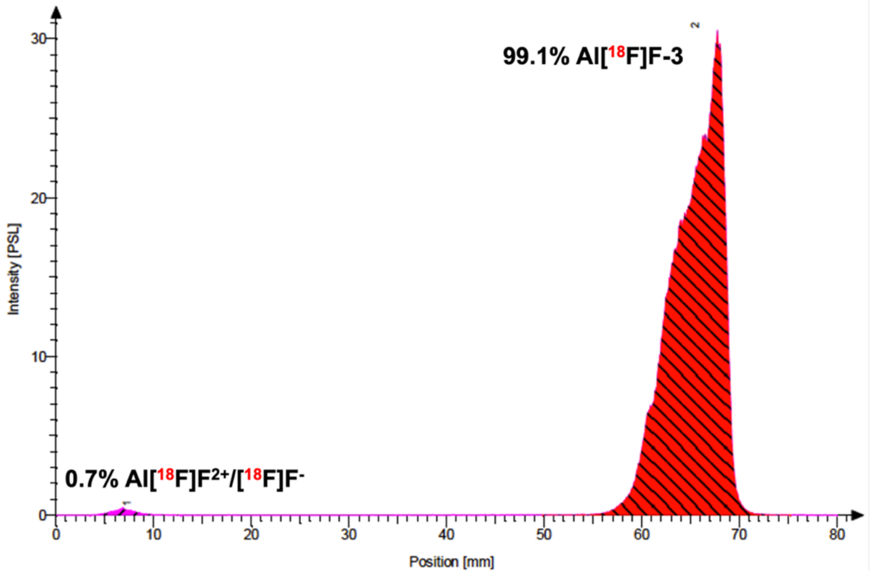

Figure S11. Radio-TLC example of a reaction mixture after 30 min: starting activity of 746 MBq and total reaction volume of 640 µL.

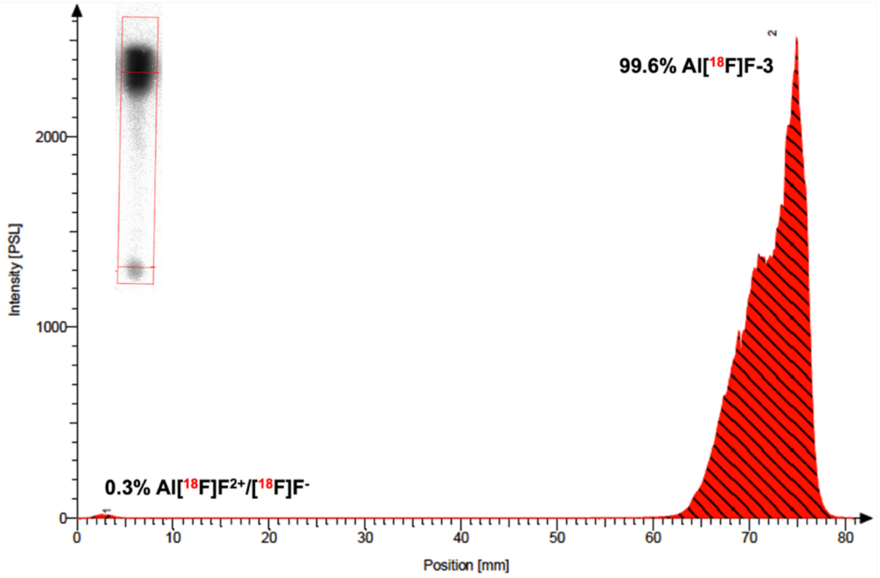

Figure S12. Example of a radio-TLC analysis of a QC sample of **Al[^{18}F]F-3** after Alox N cartridge purification.

In vitro evaluation of Al[^{18}F]F-3

Radiolabel stability (Figure 2 and Figure S13)

A sample of purified **Al[^{18}F]F-3** (17–39 MBq) was diluted in the selected media, and samples were taken at 30, 60, 90, and 120 min and directly analyzed with radio-HPLC and/or radio-TLC. The radiolabel stability of **Al[^{18}F]F-3** was tested in 1×HBSS (pH 7.2) at 37 ºC (n = 2), 5% EtOH in 1×PBS (formulation, pH 7.4) at rt (n = 2), in 80–89% mouse serum at 37 ºC (n = 2), complete medium (n = 1), and 50:50 v/v (0.1% TFA) in mQ: ACN (pH 2–2.5) at rt (n = 2) and 40 ºC (n = 2). For the stability in 80-89% mouse serum, 4×10 µL aliquots of **Al[^{18}F]F-3** were diluted with 100 µL of mouse serum in 1.5-mL microtubes and incubated for 30, 60, and 90 min. At the designated time point, 200 µL of freezer-cold ACN was added to precipitate the proteins. The microtubes were centrifuged at room temperature (14 500 *g*, 10 min). A sample was then taken from the supernatant and analyzed using radio-HPLC and radio-TLC.

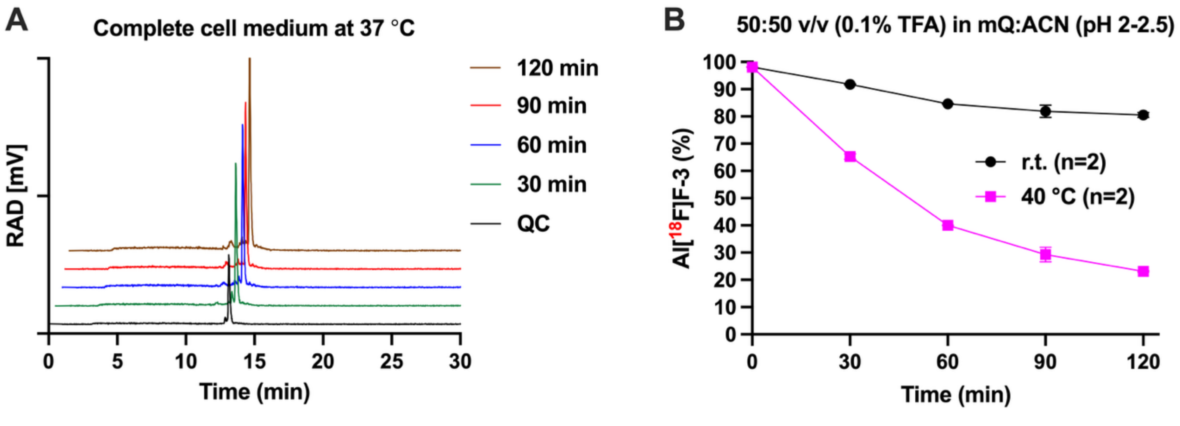

Figure S13. Additional stability graphs. **A**. Radio-HPLC analysis of the stability of **Al[^{18}F]F-3** in complete cell medium. Quality control (QC, 0 min) sample in black (retention time 14.2 min) and stability samples collected at predetermined time points (colored). **B**. Radio-TLC analysis of

Al[^{18}F]F-3 in 50:50 v/v (0.1% TFA) in mQ: ACN (pH 2–2.5) at rt (black) and 40 ºC (pink) over 120 min.

Lipophilicity

A sample of purified **Al[^{18}F]F-3** (1–2.3 MBq, n = 2) was first diluted in 2500 µL 0.01 M 1×PBS (pH 7.4) and aliquoted (500 µL, n = 4) into 1.5-mL microcentrifuge tubes containing 500 µL of 1-octanol to obtain a 1:1 (v/v) 1×PBS pH 7.4:octanol mixture. The samples were incubated at rt and vortexed every 10 min for 1 h and centrifuged (14 000 *g*, 10 min). Aliquots of 400 µL were taken from each phase and measured with a γ-counter. The lipophilicity of **Al[^{18}F]F-3** was determined with the shake-flask method as the distribution coefficient (logD) between aqueous phase represented by 0.01 PBS (pH 7.4) and 1-octanol ($LogD_{pH7.4}$), according to the equation:

$$\mathrm{LogD_{pH7.4}} = \log\left[\left(\frac{\text{counts in octanol phase}}{\text{counts in aqueous phase}}\right)\right]$$

Molar activity

The apparent A_m was calculated using the following equation:

$$\text{apparent } A_m = \frac{\text{Activity in GBq}}{\text{Amount of } \mathbf{Al[^{18}F]F-3+\ 3}}$$

A standard curve was constructed by plotting known concentrations of compound **3** corresponding to 0.15 – 17 µg ($R^2 = 0.998$). After the radiosynthesis, a sample of **Al[^{18}F]F-3** with a known activity was stored at -20 °C. The sample was injected into HPLC after decay, and the absorbance at 534 nm was used to calculate the apparent total amount of Tz (**Al[^{18}F]F-3** + **3**) present in the sample.

In vitro bioorthogonal ligations (Figure 2 and Figures S14–S16)

The in vitro SPAAC-based ligation was performed by mixing 30 nmol of **1** with 30 nmol of Ac_4ManNAz in 200 µL 1×PBS (pH 7.4) as shown in **Scheme S2A**. The reaction mixture was incubated at 37 °C for 60 min and analyzed by HPLC (method A). For the Tz-TCO ligations, (**Scheme S2B**), 120 nmol of **3** and 120 nmol of **1** were mixed in 300 µL 1×PBS (pH 7.4) and incubated at 37 °C for 30 min. The reaction mixture was analyzed by HPLC (method **A**) and LC-MS (method **C**). In addition, 9.8 MBq (apparent 1 nmol) of **Al[^{18}F]F-3** and 60 nmol of **1** were mixed in 100 µL 1×PBS (pH 7.4), followed by incubation at 37 °C for 30 min. The reaction progress was monitored by HPLC (method **A, Figures S14–S15**).

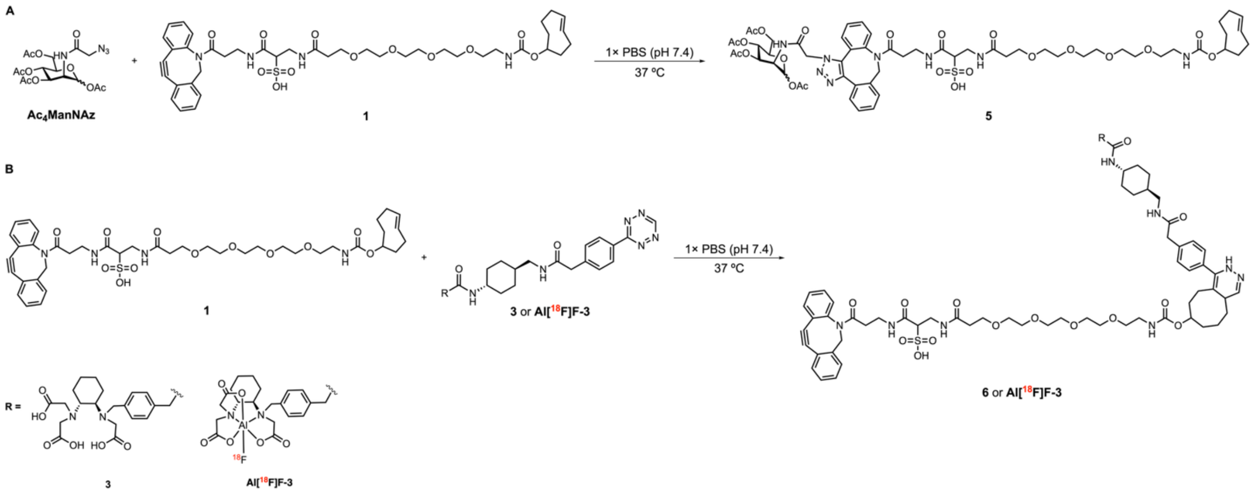

Scheme S2. **A**. SPAAC-based ligation between Ac_4ManNAz and **1** (only one possible regioisomer shown). **B**. Tz-TCO ligation between **1** and **3**/**Al[^{18}F]F-3**.

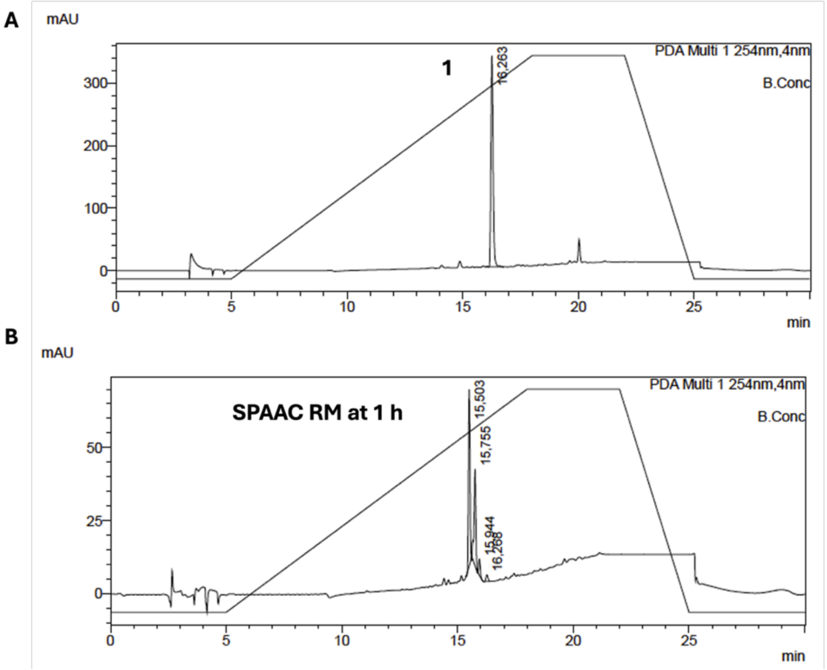

Figure S14. HPLC analysis of **1** (**A**) and SPAAC-based ligation reaction mixture after 30 min (**B**) at 254 nm.

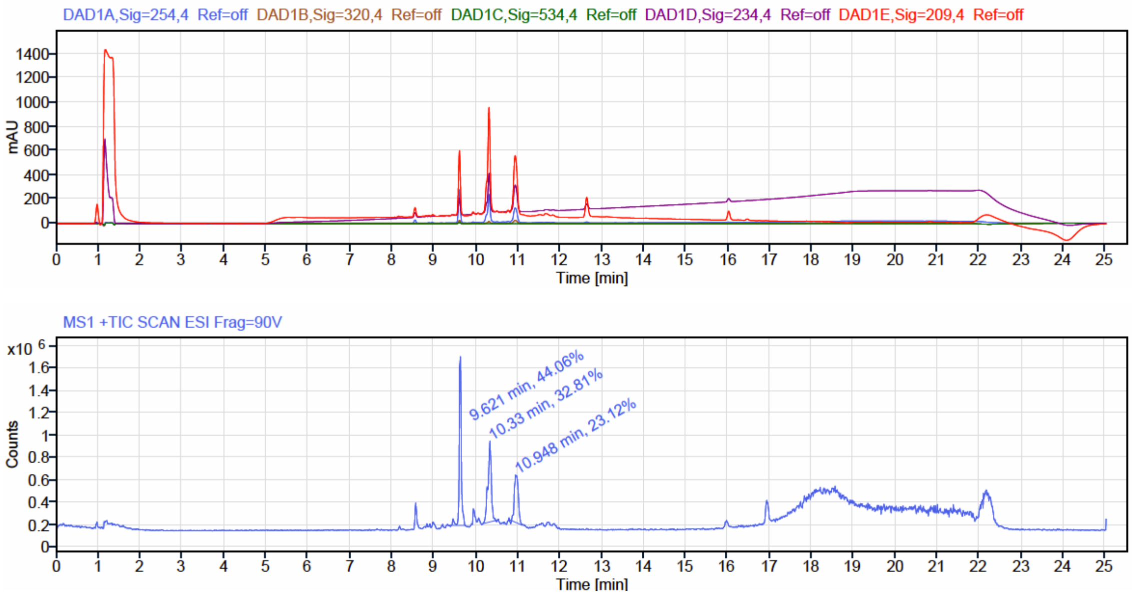

Figure S15. LC-MS analysis of the Tz-TCO ligation reaction mixture between **1** and **3** after 30 min.

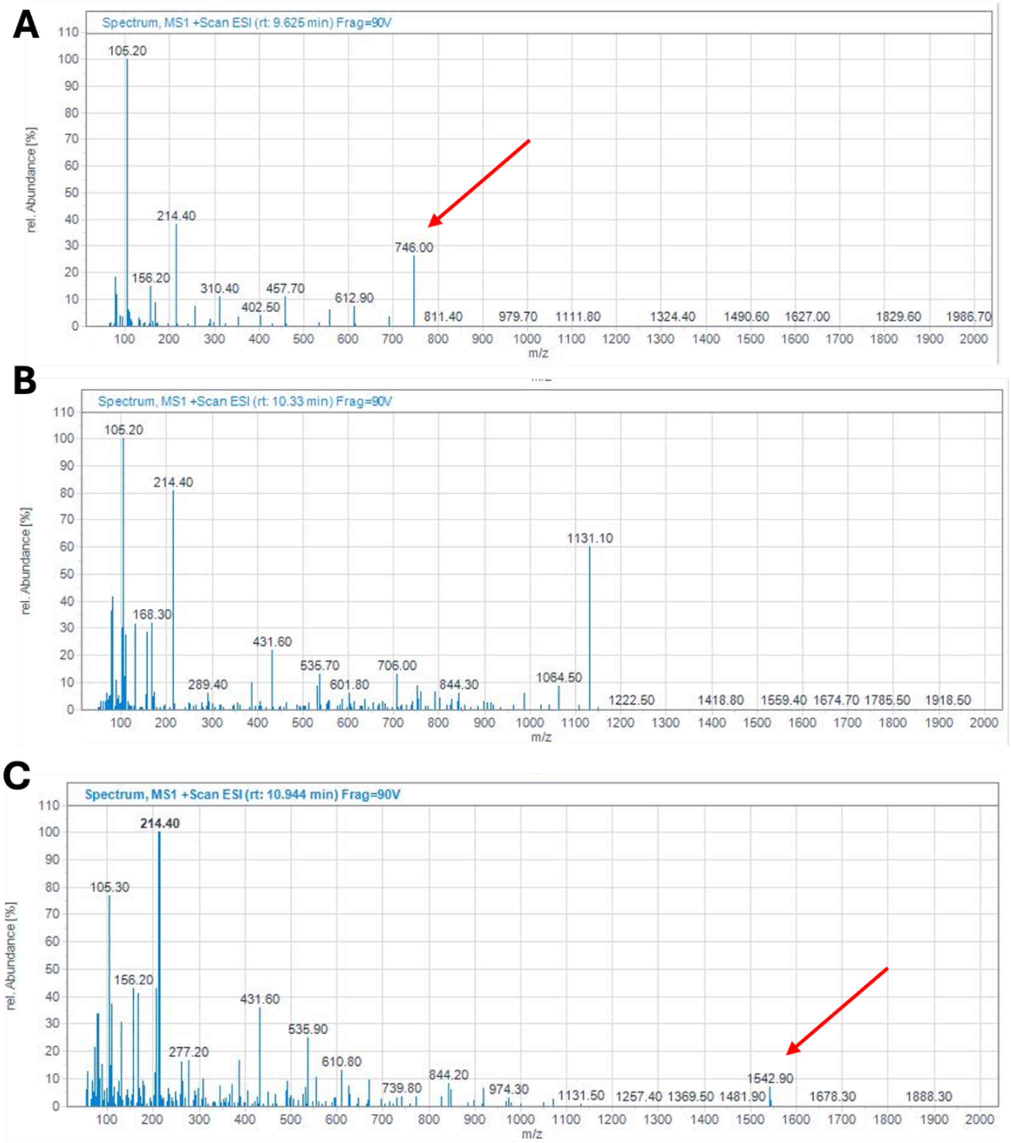

Figure S16. Extracted MS spectra of the LC-MS analysis of the Tz-TCO ligation mixture between **1** and **3**.

Cell culturing

Reagents and materials

Human Jurkat T lymphoblasts (Clone E6-1) were purchased from American Type Culture Collection (ATCC, TIB-152™, Manassas, VA, USA). T25 and T75 cell culturing flasks and non-treated well plates were purchased either from Thermo Fisher Scientific (Waltham, MA, USA), Avantor (Radnor, PA, USA) or Corning (Corning, NY, USA). Dulbecco's phosphate buffer saline (10× DPBS), Roswell Park Memorial Institute 1640 medium (RPMI-1640), GlutaMAX (100×), fetal bovine serum (FBS), Penicillin-Streptomycin (10,000 U/ml), and TrypLETM (1×) were purchased from Gibco (Life Technologies Gibco, Carlsbad, CA, USA). All culturing media were supplemented with 10% FBS, 1×GlutaMAX, and 1% PS and aseptically filtered through a sterilized 0.22 µm filter unit before use. Cell number and viability were measured using EVE™ automatic cell counter (NanoEnTek, Seoul, Korea).

Flow cytometry

Jurkat cells were incubated in a non-treated flat-bottom, polystyrene 6-well plate at 37 ºC in a humidified incubator with 5% CO_2 atmosphere. Flow cytometry analysis was done using an LSRFortessa (BD Biosciences, Franklin Lake, NJ, USA). Data were analyzed using FlowJo software (version v10.10.0, Tree Star, Ashland, OR, USA).

Protocol 1: evaluation of DBCO-PEG$_2$-TCO

Jurkat cells were incubated in triplicate (n = 3) with either 50 µM of Ac$_4$ManNAz or 0.5% (v/v) DMSO in complete medium (0.35×10^6 in 1.2 mL per well) for 72 h at 37 ºC. After incubation,

cells were transferred to 1.5-mL microtubes, centrifuged at 4 °C (10 *g*, 5 min), and washed twice with 500 µL of fluorescence-activated cell sorting (FACS) buffer (2% FBS in 1×DPBS). During each wash, the supernatant was discarded, and the cell pellet was resuspended in fresh buffer prior to centrifugation. Cell number and viability were determined, after which cells were resuspended in 500 µL of either 1×HBSS (pH 7.2) or 10 or 20 µM of DBCO-PEG$_2$-TCO in 1×HBSS and incubated on a 360-degree tube rotator (at a fixed speed of 20 RPM, Avantor, Radnor, PA, USA) for 60 min at rt. After incubation, cells were centrifuged and washed twice with 500 µL of FACS buffer, then incubated with either 100 µL of 10 µM sulfo-Cy3-Tz in FACS buffer (freshly prepared) or 100 µL of FACS buffer at 37 °C for 30 min. Subsequently, 400 µL of FACS buffer was added, and the cells were washed twice with 500 µL of the same buffer. Cells were then resuspended in 500 µL of FACS buffer at a concentration of 1×10^6 cells/mL and transferred to FACS tubes equipped with 35-µm cell strainer caps. Selected samples were stained with 10 µL of live/dead stain 7-aminoactinomycin D (7-AAD, Miltenyi Biotec, Bergisch Gladbach, Germany) for 10 min at rt in the dark prior to analysis.

Protocol 2: evaluation of compound 1

Jurkat cells were seeded and incubated with either 50 µM of Ac$_4$ManNAz or 0.5% (v/v) DMSO in complete medium (0.5×10^6 in 1.5 mL per well) for 72 h at 37 ºC. After incubation, cells that had received the same treatment were combined, centrifuged at 4°C (130 *g*, 5 min), and washed twice with 5 mL of 1×DPBS. During each washing step, the supernatant was discarded, and the cell pellet was resuspended in fresh buffer before the next centrifugation. Cell number and viability were measured, and cells were resuspended in triplicate (n = 3; 2×10^6 cells in 500 µL) in either 1×DBPS or 10 µM of compound **1** in 1×DPBS and incubated on a tube rotator placed inside an

incubator at 37 °C for 60 min. After incubation, cells were centrifuged at 4 °C (10 *g*, 5 min), washed twice with 500 µL of FACS buffer, and then incubated in 100 µL of 1 or 10 µM of sulfo-Cy3-Tz solution in FACS buffer—or 100 µL of FACS buffer—on a tube rotator placed inside an incubator at 37 °C for 30 min. Subsequently, 400 µL of FACS buffer was added, followed by two additional washes with 500 µL of the same buffer. Finally, cells were resuspended in 500 µL of FACS buffer and transferred to FACS tubes equipped with 35 µm cell strainer caps. Selected samples were stained with 10 µL of live/dead cell stain 7-AAD for 10 min at rt in the dark prior to analysis.

Flow cytometry analysis was performed by sequential gating on lymphocytes, followed by singlet discrimination and assessment of Cy3 signal in the single cell gate. Identical gating strategies were applied across all samples. 7-AAD was used to assess the viability of the cells in the selected samples. Notably, dead cells were largely excluded during the initial lymphocyte gate, and no substantial differences in viability were observed between samples.

Flow cytometry results: evaluation of DBCO-PEG$_2$-TCO

The commercially available DBCO-PEG$_2$-TCO derivative produced substantial fluorescence in both control and Ac$_4$ManNAz-treated cells (**Figure S17)**, indicating prominent nonspecific membrane association, consistent with its relatively high lipophilicity. Notably, a higher fraction of Cy3-positive cells was observed in controls (>99%) than in Ac$_4$ManNAz-treated cells (71.9–83.6%). However, within the Cy3-positive population, Ac$_4$ManNAz-treated cells exhibited markedly higher MFI with values approximately 2–3-fold greater than controls (**Supporting Table S2**). This pattern suggests that, despite nonspecific binding, the IEDDA

reaction (and thus productive TCO presentation) was more efficient in metabolically engineered cells. Increasing the concentration of DBCO-PEG$_2$-TCO increased fluorescence intensity in both groups, with a larger effect in Ac$_4$ManNAz-treated cells, consistent with higher azide density following MGE. In contrast, incubation with sulfo-Cy3-Tz alone resulted in minimal labeling in both control and Ac$_4$ManNAz-treated cells (2.8–9.1% Cy3-positive), and increasing the Tz concentration had only a minor effect (**Figure S17B**).

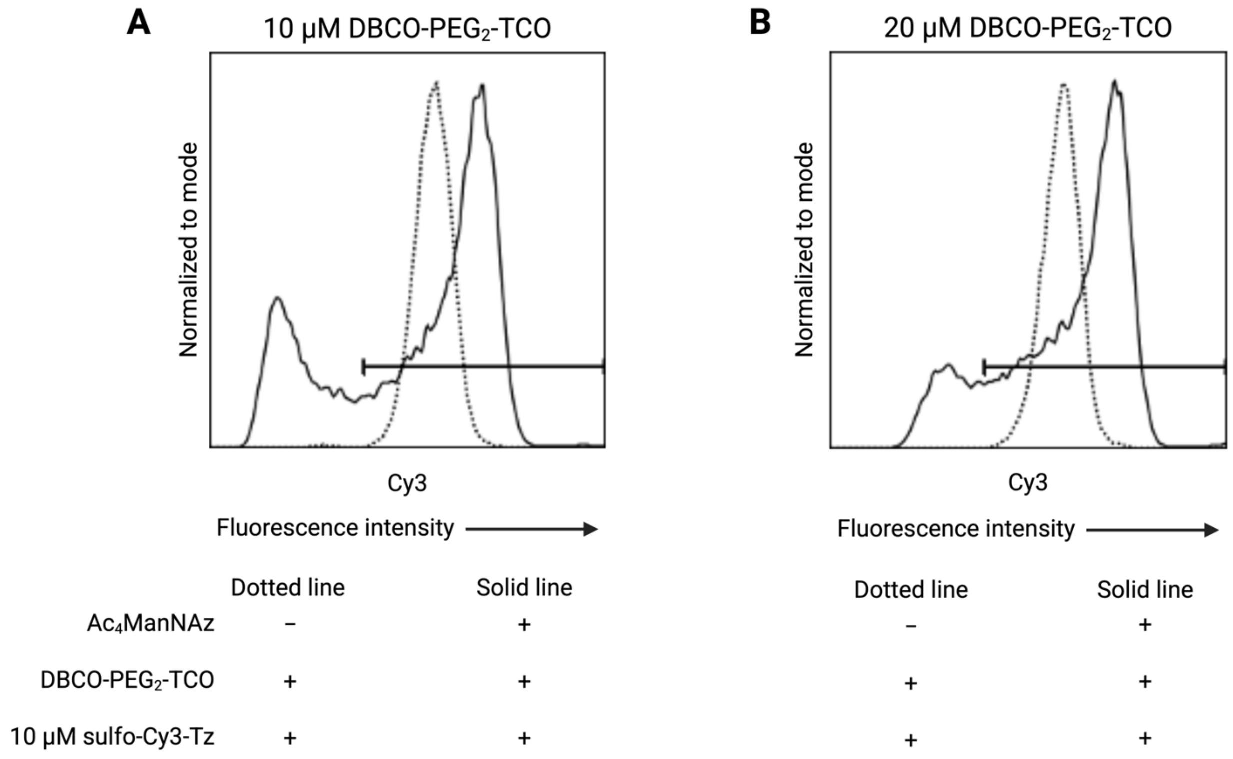

Figure S17. Flow cytometry analysis of two-step cell-surface labeling using 10 μM DBCO-PEG$_2$-TCO (**A**) or 20 μM DBCO-PEG$_2$-TCO (**B**). Jurkat cells were treated with 0.5% v/v DMSO (control, dotted lines) or Ac$_4$ManNAz (solid lines) for 72 h, followed by SPAAC-based ligation with DBCO-PEG$_2$-TCO and subsequent labeling with sulfo-Cy3 Tz (10 μM) via the Tz-TCO ligation. Histograms are representative examples from a single sample per condition.

Table S2. Quantitative flow cytometry analysis following two-step labeling using **Protocol 1** expressed as percentage of Cy3-positive cells and median fluorescence intensity (MFI) for samples shown in **Figure 3A**.

**Sample**	**Treatment 1 (72 h)**	**Treatment 2**	**Treatment 3**	**% of Cy3-positive cells**	**MFI of Cy3-positive cells**
**Control 1**	0.5% DMSO	10 µM DBCO-PEG2-TCO	10 µM sulfo-Cy3 Tz	99.2	1239
**Control 2**	0.5% DMSO	20 µM DBCO-PEG2-TCO	10 µM sulfo-Cy3 Tz	99.7	1532
**Treated 1**	$Ac_4ManNAz$	10 µM DBCO-PEG2-TCO	10 µM sulfo-Cy3 Tz	71.9	3470
**Treated 2**	$Ac_4ManNAz$	20 µM DBCO-PEG2-TCO	10 µM sulfo-Cy3 Tz	83.6	4632

Table S3. Quantitative flow cytometry analysis following two-step labeling using **Protocol 2** expressed as percentage of Cy3-positive cells and median fluorescence intensity (MFI) for samples shown in **Figure 3B**.

**Sample**	**Treatment 1 (72 h)**	**Treatment 2**	**Treatment 3**	**% of Cy3-positive cells**	**MFI of Cy3-positive cells**
**Control 1**	0.5% DMSO	1×PBS	1 µM sulfo-Cy3 Tz	2.79	924
**Control 2**	0.5% DMSO	1×PBS	10 µM sulfo-Cy3 Tz	9.13	1239
**Control 3**	0.5% DMSO	10 µM **1**	1 µM sulfo-Cy3 Tz	2.85	1048
**Control 4**	0.5% DMSO	10 µM **1**	10 µM sulfo-Cy3 Tz	6.04	1246
**Treated cells - 1**	$Ac_4ManNAz$	1×PBS	1 µM sulfo-Cy3 Tz	2.58	1063
**Treated cells - 2**	$Ac_4ManNAz$	1×PBS	10 µM sulfo-Cy3 Tz	6.77	1182
**Treated cells - 3**	$Ac_4ManNAz$	10 µM **1**	1 µM sulfo-Cy3 Tz	99.1	2669
**Treated cells - 4**	$Ac_4ManNAz$	10 µM **1**	10 µM sulfo-Cy3 Tz	99.4	5114

In vitro cell radiolabeling

The 72-h incubation of Jurkat cells was done in non-treated, flat-bottom, polystyrene 6-well plates at 37 ºC in a humidified incubator with 5% CO_2 atmosphere.

Protocol 1: initial experiments

Jurkat cells were incubated with either 50 µM of Ac_4ManNAz or 0.5% (v/v) DMSO in complete medium (0.35×10^6 in 1.2 mL per well) for 72 h at 37 ºC. After the incubation, cells from individual wells were transferred to 1.5-mL microtubes and centrifuged at 4°C (10 *g*, 5 min) and washed twice with 0.5 mL of 1×HBSS. During each washing step, the supernatant was discarded, and the cell pellet was resuspended in fresh buffer before the next centrifugation.

Cells were then resuspended in a solution of either DBCO-PEG$_2$-TCO, compound **1** in 1×HBSS, or 1×HBSS (n = 3 per condition) and incubated for 30 min at different temperatures. For rt incubations, microtubes were put on a tube rotator; for 37 ºC incubations, samples were placed in an incubator equipped with an orbital shaker (50–100 rpm); and for 4 ºC incubations, microtubes were kept on ice and mixed manually every 10 min.

Following the SPAAC-based ligation, cells were centrifuged at 4 °C (10 *g*, 5 min), washed twice according to the conditions specified in **Table S4**. Cell number and viability were measured for selected samples. Cells were then resuspended in a solution of **Al[^{18}F]F-3** (≤ 5% EtOH in 1×HBSS) and incubated at 37 ºC for 30 min.

After radiolabeling, cells were centrifuged, and the supernatant (free fraction) was transferred to 6-mL Pony vials (Revvity, Waltham, MA, USA). Cells were subsequently washed twice with 1×HBSS (0.5–1 mL), and the wash fractions were combined with the free fraction.

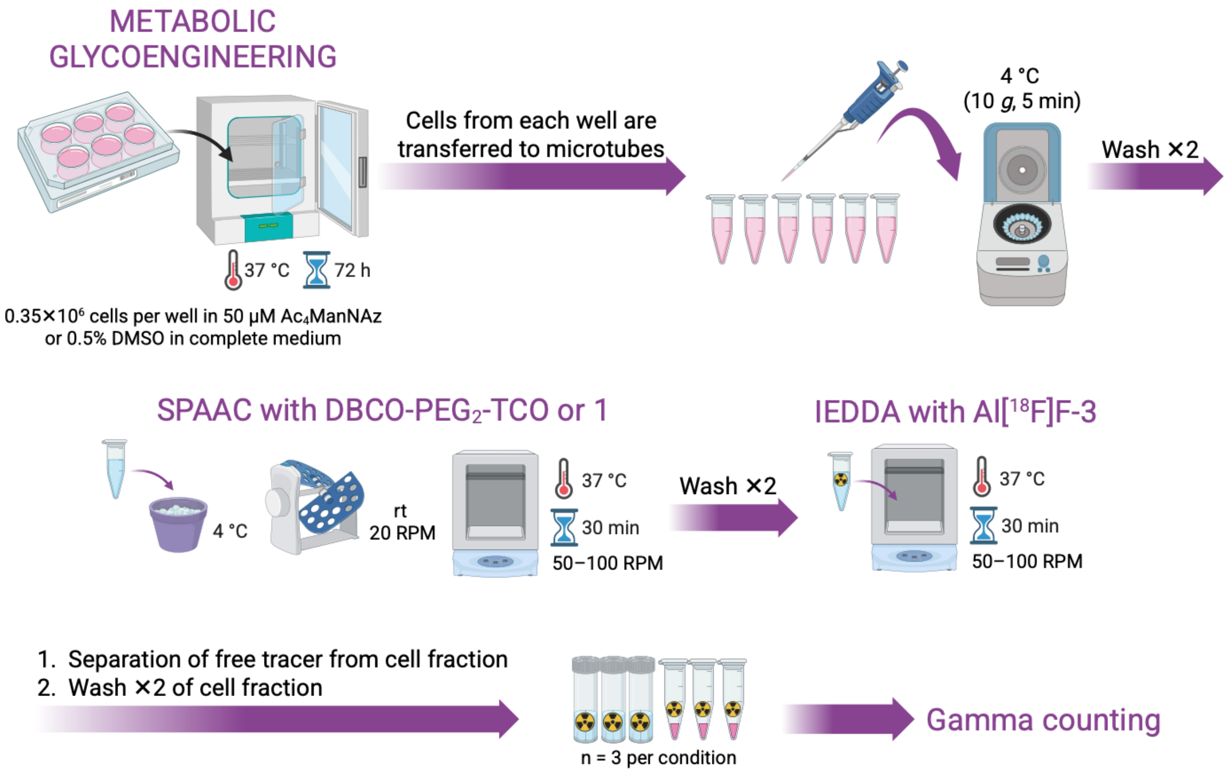

Figure S18. Schematic overview of protocol 1 for initial MGE-based cell radiolabeling experiments. Jurkat T cells were metabolically glycoengineered by incubation with 50 µM $Ac_4ManNAz$ or 0.5% v/v DMSO (control) in complete medium for 72 h at 37 ºC. Then after, cells from each well were transferred individually to microtubes, washed twice, and incubated with DBCO-PEG_2-TCO or compound **1** for the SPAAC-based ligation in different conditions (4ºC, rt, or 37 ºC, 30 min). After two washes, cells underwent Tz-TCO ligation with the fluorine-18-labeled Tz **Al[^{18}F]F-3 (**37 ºC, 30 min). Following IEDDA, the supernatant (free fraction) was collected and combined with two subsequent washes. Cell pellets and free fractions were measured separately by gamma counting. Created in BioRender. Biti, A. (2026) https://BioRender.com/7ohlg32

Standards for **Al[^{18}F]F-3** were prepared by diluting one dose of the **Al[^{18}F]F-3** formulation and transferring an appropriate volume into 6-mL Pony vials (n = 3). All fractions and standards were measured using a γ-counter, and results were normalized to the corresponding standard. Detailed experimental conditions are summarized in **Table S4**.

Table S4. Summary of experimental conditions evaluated for cell radiolabeling under **Protocol 1**. The incubation with **Al[^{18}F]F-3** was done at 37 ºC for 30 min.

**Entry**	**Incubation conditions for SPAAC-based ligation**	**Washing after SPAAC-based ligation**	**Al[^{18}F]F-3**
**a**	10 µM of DBCO-PEG$_2$-TCO in 1×HBSS at rt, 30 min, n = 3	1×HBSS ×3	0.5 MBq
**b**	10 µM of DBCO-PEG$_2$-TCO in 1×HBSS at 4 ºC, 30 min, n = 3	1×HBSS ×3	0.5 MBq
**c**	10 µM of **1** in 1×HBSS at rt, 30 min, n = 3	1×HBSS ×2	0.5 MBq
**d**	10 µM of **1** in 1×HBSS at 37 ºC, 30 min, n = 3	1× HBSS ×2	0.5 MBq
**e**	10 µM of **1** in 1×HBSS at rt, 30 min, n = 3	PBS-T ×1 + 1×HBSS ×1	0.5 MBq
**f**	-	1×HBSS ×2	0.5 MBq
**g**	10 µM of **1** in 1×HBSS at 37 ºC, 30 min, n = 3	1×HBSS ×2	0.5 MBq
**h**	10 µM of **1** in 1×HBSS at 37 ºC, 30 min, n = 3	PBS-T ×2	0.5 MBq
**i**	10 µM of **1** in 1×HBSS at 37 ºC, 30 min, n = 3	1×HBSS ×2	0.1 MBq
**j**	10 µM of **1** in 1×HBSS at 37 ºC, 30 min, n = 3	1×HBSS ×2	0.5 MBq
**k**	10 µM of **1** in 1×HBSS at 37 ºC, 30 min, n = 3	1×HBSS ×2	2.0 MBq

Protocol 2: optimized conditions

Jurkat cells were incubated with either 50 µM of Ac$_4$ManNAz or 0.5% (v/v) DMSO in complete medium (0.5×10^6 in 1.5 mL per well) for 72 h at 37 ºC. After incubation, cells corresponding to each condition were combined, centrifuged at 4°C (130 *g*, 5 min), and washed twice with 1×DPBS (5 mL). Cell number and viability were determined, after which cells were resuspended at various concentrations (2–10×10^6 in 0.5–1 mL) in 10 µM of **1** in 1×DPBS or 1×DPBS and transferred into 1.5-mL microtubes (n = 3–7 per condition). Samples were incubated on a tube rotator placed inside an incubator at 37 ºC for 60 min. Following the SPAAC-based ligation, cells were centrifuged at

4°C (10 *g*, 5 min), washed twice with 1×DPBS (0.5–1 mL, depending on the cell number), and subsequently incubated with **Al[^{18}F]F-3** (≤ 5% EtOH in 1×DPBS) at 37 ºC for 30 min.

After radiolabeling, microtubes were centrifuged, and the supernatant (free fraction) was transferred to a 6 mL Pony vial. Cells were then washed twice with 1×DPBS (0.5–1 mL), and the wash fractions were combined with the free fraction. In entries **e** and **f**, **Table S5**, free fractions were combined into a single 15-mL Falcon tube, while cell fractions were combined into a single 1.5-mL microtube. Experimental conditions are summarized in **Table S5**.

Standards for **Al[^{18}F]F-3** (for entries **a**–**d**, **Table S5**) were prepared by diluting one dose of the formulation and pipetting an appropriate volume into Pony vials (n = 3). All fractions and standards for entries **a**–**d** were measured first with a dose calibrator and then with a γ-counter. Cell number and viability were measured for the samples exhibiting the highest activity in entries **b**–**d**, and for the combined cell fraction in entries **e** and **f** (**Table S5**).

Table S5. Summary of experimental conditions evaluated for cell radiolabeling under **Protocol 2**. The incubation with **Al[^{18}F]F-3** was done at 37 ºC for 30 min.

**Entry**	**Conditions for SPAAC-based ligation**	**Al[^{18}F]F-3**
**a***	2×10^6 cells /0.5 mL, n = 3	1.0 MBq
**b***	2×10^6 cells /0.5 mL, 10 μM of **1** in 1×PBS at 37 ºC, 60 min, n = 3	1.0 MBq
**c***	4×10^6 cells /0.5 mL, 10 μM of **1** in 1×PBS at 37 ºC, 60 min, n = 3	1.0 MBq
**d***	4×10^6 cells /0.5 mL, 10 μM of **1** in 1×PBS at 37 ºC, 60 min, n = 3	5.0 MBq
**e**	6×10^6 cells /1 mL, 10 μM of **1** in 1×PBS at 37 ºC, 60 min, n = 3	5.0 MBq
**f**	6×10^6 cells /1 mL, 10 μM of **1** in 1×PBS at 37 ºC, 60 min, n = 3	7.5 MBq
**g**	6×10^6 cells /1 mL, 10 μM of **1** in 1×PBS at 37 ºC, 60 min, n = 3	10.0 MBq
**h**	10×10^6 cells /1 mL, 10 μM of **1** in 1×PBS at 37 ºC, 60 min, n = 3	5.0 MBq
**i**	10×10^6 cells /1 mL, 10 μM of **1** in 1×PBS at 37 ºC, 60 min, n = 3	10.0 MBq
**j**	4×10^6 cells /0.5 mL, 10 μM of **1** in 1×PBS at 37 ºC, 60 min, no control, n = 6	10 MBq
**k**	4×10^6 cells /0.5 mL,10 μM of **1** in 1×PBS at 37 ºC, 60 min, no control, n = 7	7.0 MBq

*For experiments in entries **a**–**d**, empty microtubes were also treated with **1** and/or **Al[^{18}F]F-3** under the same conditions as the cell samples.

The cell-associated activity (%) was calculated according to the equation:

$$\text{cell} - \text{associated activity} = \frac{\text{average counts of replicates}}{\text{average counts of standard replicates}} \times 100$$

Additional experiments were conducted using a similar protocol. Jurkat cells were incubated with either 40 μM of $Ac_4ManNAz$ or 0.5% (v/v) DMSO in complete medium. The SPAAC-based ligation was performed with 1×10^6 cells/0.5 mL per sample. After Tz-TCO ligations, cells were either kept in the same microtubes or transferred to Spin-X Centrifuge Tube Filters (Corning) for washing (at 4°C, 14 000 *g*, 10 min). When cells were retained in microtubes throughout the Tz-TCO ligations and subsequent washings, higher apparent cell-associated activity was measured compared to cells transferred to centrifugal filters for washing. Filter-based separation markedly reduced nonspecific activity, providing a more accurate estimate of cell-associated activity. Increasing the concentration of compound **1** from 10 μM (**Figure S17A**) to 20 μM (**Figure S17B**) did not enhance labeling efficiency, indicating that the reaction had reached a plateau under these conditions. In contrast, microtube-associated activity increased substantially (1.3±0.7% vs 9.0±0.9%), consistent with increased nonspecific adsorption of the radiolabeled compound to the plastic surface.

Cell labeling results

Protocol 1

Cell radiolabeling using the commercially available DBCO-PEG_2-TCO (10 μM, rt or 4 ºC for 30 min) was evaluated only once using Protocol 1 (**Figure S18**). Following the Tz-TCO ligation with 0.5 MBq of **Al[^{18}F]F-3**, cell-associated activity was higher in control cells than in $Ac_4ManNaz$-treated cells at both rt (15.1±4.4% vs 10.0±2.6%) and 4 ºC (17.2±0.3% vs 9.1±0.5%), showing

that lowering the incubation temperature for the SPAAC-based ligation does not improve selective cell-surface radiolabeling under these conditions.

Given concerns about the lipophilicity of the DBCO moiety and its potential nonspecific membrane association, we investigated post-SPAAC washing conditions following incubation with compound **1** (10 µM at rt or 37 ºC for 30 min, **Figure S19B–C**). Washing twice with PBS containing 0.05% Tween 20 (PBS-T, pH 7.4) or using a combination of PBS-T and 1×HBSS did not meaningfully change cell-associated activity compared with washing with 1×HBSS alone. Notably, across all tested conditions, cell-associated activity was consistently higher in Ac_4ManNAz-treated cells than in control cells, indicating improved specificity relative to DBCO-PEG_2-TCO. Cells exposed to **Al[^{18}F]F-3** alone exhibited minimal associated activity (0.6±0.1% for control cells and 0.5±0.1% for Ac_4ManNAz-treated cells), supporting negligible nonspecific binding of **Al[^{18}F]F-3** to the cell surface (**Figure S19C**).

We then investigated the effect of starting activity on Tz-TCO ligation and overall radiolabeling efficiency (**Figure S19D**). Increasing the activity from 0.1 to 2 MBq resulted in a progressive decrease in cell-associated activity, which may reflect a combination of limited TCO availability on the cell surface and activity-dependent effects at higher radioactivity concentrations. Across the range tested, Ac_4ManNAz-treated cells consistently exhibited higher cell-associated activity than controls. The difference was more pronounced at the lowest activity (0.1 MBq), suggesting that Tz-TCO ligation contributes most effectively under these conditions. At 0.5 MBq, the difference was reduced, and at 2 MBq it was minimal.

Live cell number and viability were measured at the end of labeling for a single replicate per condition and showed variability in both viable cell number (0.3–1.3×10^6) and viability (66–92%).

Because all samples were seeded at the same initial cell density, these differences likely reflect variability in cell growth during the 72-h incubation and/or differential effects of the incubation with DBCO-PEG$_2$-TCO or compound **1** in control versus Ac$_4$ManNAz-treated cells. As cell number and viability were not measured for all replicates, these parameters were not used for normalization. Instead, radiolabeling results were normalized to the added activity of **Al[^{18}F]F-3** using counting standards.

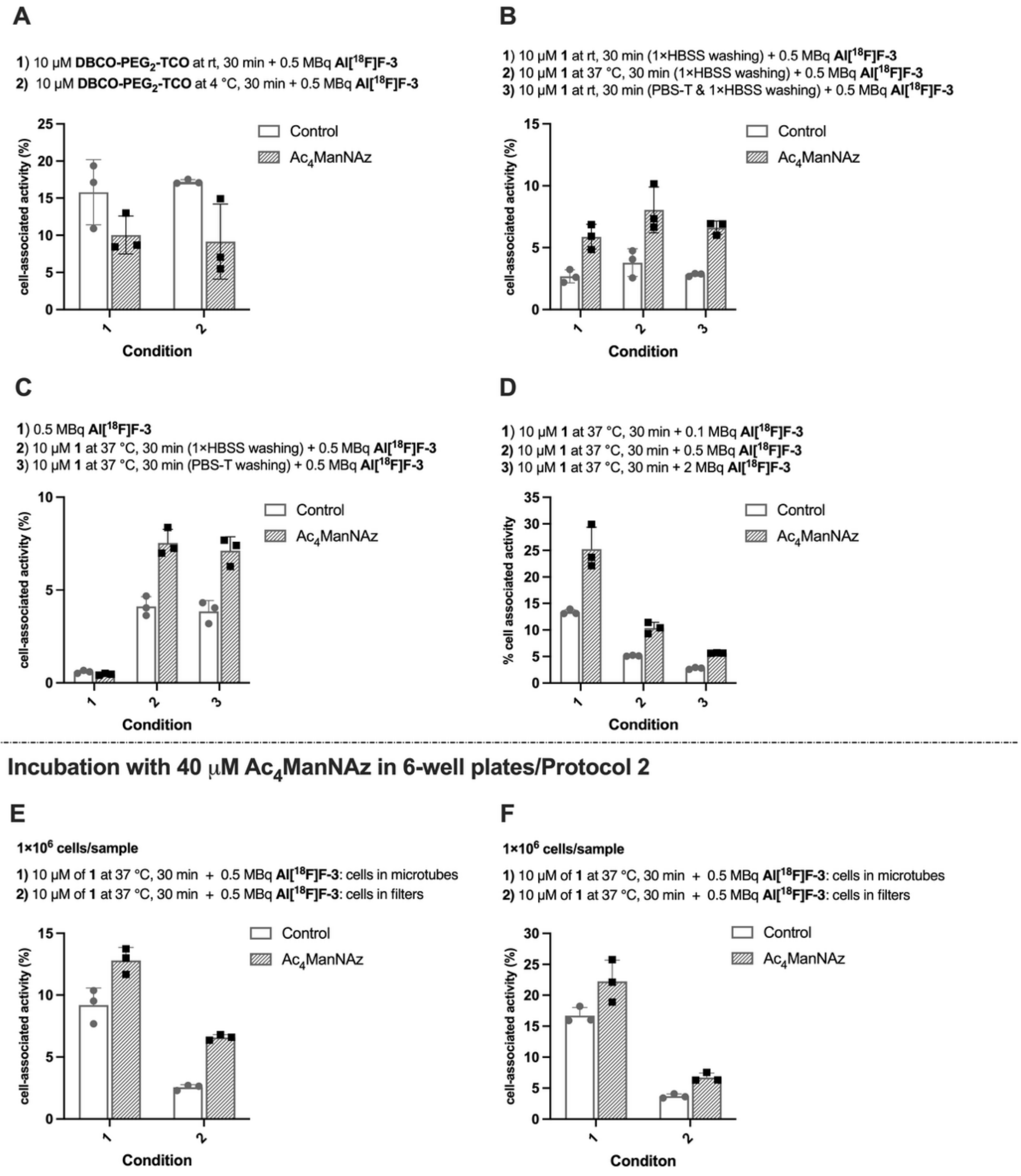

Figure S19. Cell-associated activity (%) of control and metabolically glycoengineered Jurkat cells following SPAAC-based and Tz-TCO ligations under different conditions. **A**. Comparison of SPAAC-based ligation carried out at rt and 4 ºC using DBCO-PEG$_2$-TCO. **B–C**. Effect of washing conditions and SPAAC-based ligation temperature when using compound **1**. **D**. Effect of **Al[^{18}F]F-3** starting activity (0.1–2 MBq) on labeling following SPAAC-based ligation with 10 µM of compound **1** at 37 ºC for 30 min. **E-F** Evaluation of filter-based separation during cell radiolabeling to reduce nonspecific activity. Cell-associated activity is shown for control and Ac$_4$ManNAz-treated cells when cells were either kept in microtubes or transferred to centrifugal filters after the Tz-TCO ligation. Results are presented as mean±SD (n = 3 technical replicates).

Animal studies

The studies were carried out in healthy female athymic nude mice (Rj:ATHYM-*Foxn1*$^{nu/nu}$, weighing 18–26 g, aged 8−25 weeks, Janvier Labs, Le Genest-Saint-Isle, Mayenne, France) housed in conventional polysulfone cages with aspen bedding (Tapvei®, Harjumaa, Estonia) and enrichments (e.g., nesting material and disposable cardboard hut). Pelleted food (Teklad 2019C diet, Envigo, Huntington, UK) and water were provided ad libitum. Conditions were maintained at 12/12 h dark/light cycle, 22±1 °C, and 55±15% relative humidity.

Cell labeling for animal studies

Jurkat cells were seeded on 6-well plates and incubated with 50 µM of Ac$_4$ManNAz in complete medium (0.5×10^6 in 1.5 mL per sample) for 72 h at 37 ºC. After MGE, azide-modified cells were combined and washed twice with 5 mL of 1×DPBS. Then, azide-modified cells were resuspended in an appropriate volume of 1×DPBS, and cell number and viability were determined.

For pretargeting experiments and in vitro radiolabeling, azide-modified cells were resuspended in a solution of 10 μM of compound **1** in 1×DPBS and incubated in microtubes (4×10^6 in 0.5 mL, n = 7–8) for 1 h at 37 °C (on a tube rotator placed inside an incubator). After the SPAAC-based ligation, cells were washed twice with 500 μL of 1×DPBS, combined in a single microtube, and resuspended in an appropriate volume of 1×DPBS. Cell number and viability were reassessed. The obtained TCO-modified cells were injected iv for pretargeting experiments or further treated with **Al[^{18}F]F-3** for in vitro labeling.

For in vitro radiolabeling, TCO-modified cells were incubated with 7 MBq of **Al[^{18}F]F-3** (4×10^6/0.5 mL ≤ 5% EtOH in 1×DPBS) for 30 min at 37 °C. Finally, in vitro fluorine-18-labeled cells were washed twice with 500 μL of 1×DPBS, combined in a single microtube, and resuspended in an appropriate volume of 1×DPBS. Cells were kept on ice and gently resuspended by pipetting prior to injection.

In vivo evaluation

Al[^{18}F]F-3 (1.49±0.18 MBq in 200 μL ≤5.7% EtOH in 1×DPBS, n = 5) and in vitro fluorine-18-labeled cells (0.16±0.02 MBq, 2.5×10^6 in 100–150 μL 1×DPBS, n = 4) were administered intravenously to assess the biodistribution of the free radiotracer and fluorine-18-labeled cells, respectively.

In pretargeting experiments, TCO-modified cells were injected intravenously and allowed to circulate for 30 min (2×10^6 in 100 μL 1×DPBS, n = 3) or 120 min (2.5×10^6 in 100 μL 1×DPBS, n = 3) before administration of **Al[^{18}F]F-3 (**1.91±0.73 MBq in 100 μL ≤ 5.7% EtOH in 1×DPBS).

To assess nonspecific binding, azide-modified control cells (2.5×10^6 in 100 μL 1×DPBS, n = 3) were administered intravenously under identical conditions, followed by **Al[^{18}F]F-3** after 120 min (1.71±0.04 MBq in 100 μL 8.5% EtOH in 1×DPBS).

Dynamic PET/CT imaging and ex vivo biodistribution

A dynamic whole-body 90-min PET/CT scan was acquired after fluorine-18-labeled tracer of cell administration under isoflurane anesthesia in medical oxygen carrier ($1\ \mathrm{L\ min^{-1}}$) using the Molecubes benchtop β-CUBE and X-CUBE (Molecubes NV, Gent, Belgium) for PET and micro-CT, respectively. PET/CT images were analyzed with the VivoQuant (InviCRO LLC, Needham, MA, USA, version 4.0.0), and the images of the dynamic scans were reconstructed into 6 frames ×10 sec, 4 frames ×1 min, 5 frames ×5 min, 6 frames ×10 min. The ROIs were the heart, lungs, liver, gallbladder, small intestine, spleen, kidneys, and bladder. ROIs were drawn manually on dynamic images and normalized in SUV unit to generate TAC curves, and AUC was calculated for each organ using GraphPad Prism 10 (San Diego, CA, USA) built-in analysis. At the end of the scan, animals were euthanized by cervical dislocation under anesthesia (for PET imaging group) or CO_2 asphyxiation followed by cervical dislocation (for biodistribution group). The tissues of interest were collected for γ-counting. The biodistribution results are presented as %ID/g. The injected dose for each administration was calculated by measuring syringe activity with a dose calibrator before and after the injection and decay-correcting the difference to the time of injection for PET analysis. For ex vivo biodistribution, all measured activities were decay-corrected to the start of gamma counting.

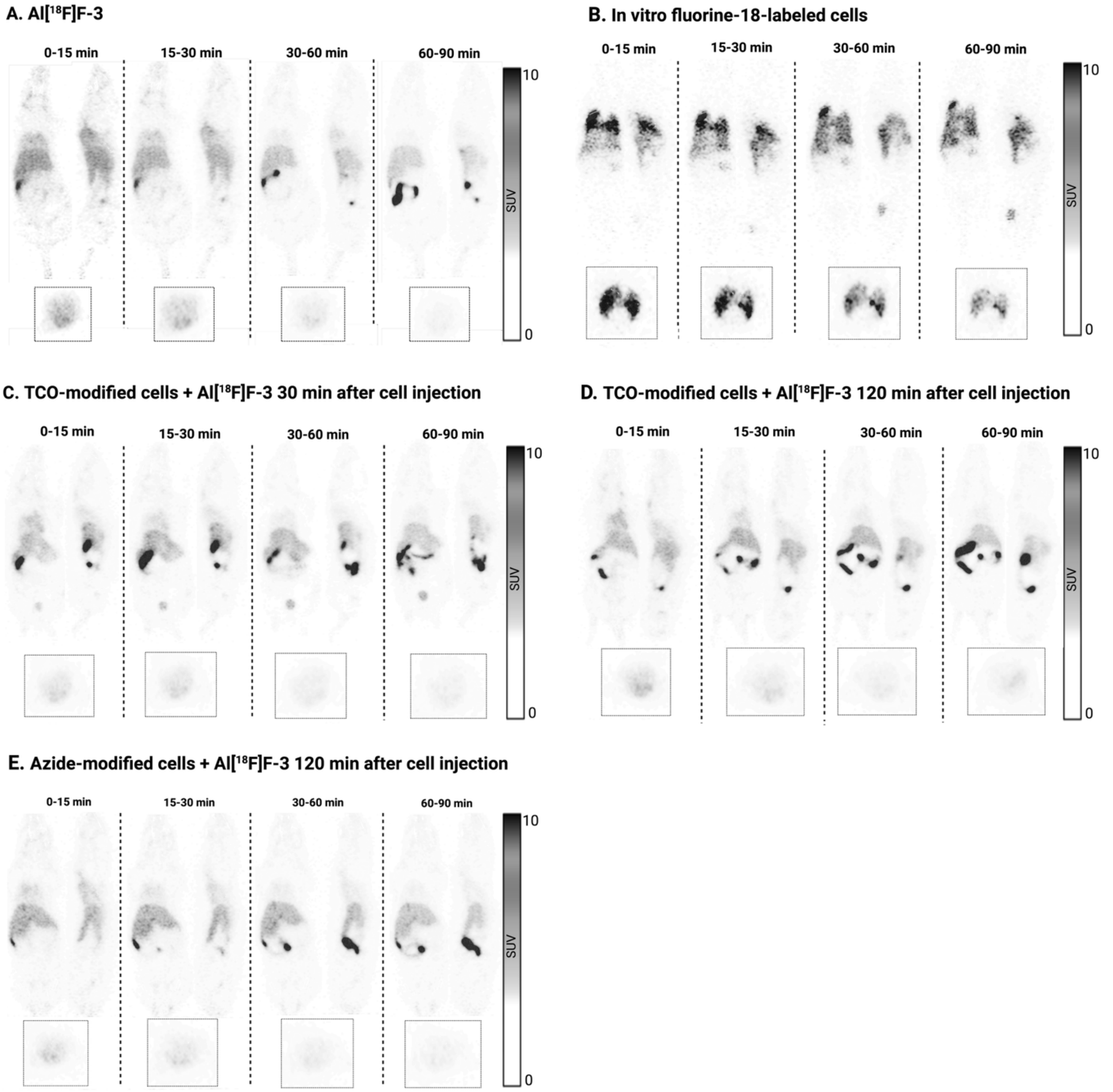

Figure S20. Representative summed PET images in grayscale (0–15, 15–30, 30–60, and 0–90 min) in coronal (left), sagittal (right), and transversal (bottom, through the lungs) planes and maximum intensity project (MIP, 0-90 min) following intravenous injection of **Al[^{18}F]F-3** (**A**), in vitro fluorine-18-labeled cells (**B**), TCO-modified cells followed by **Al[^{18}F]F-3** injection after 30 min (**c**), TCO-modified cells followed by **Al[^{18}F]F-3** injection after 120 min (**D**), and azide-modified cells followed by **Al[^{18}F]F-3** injection after 120 min.

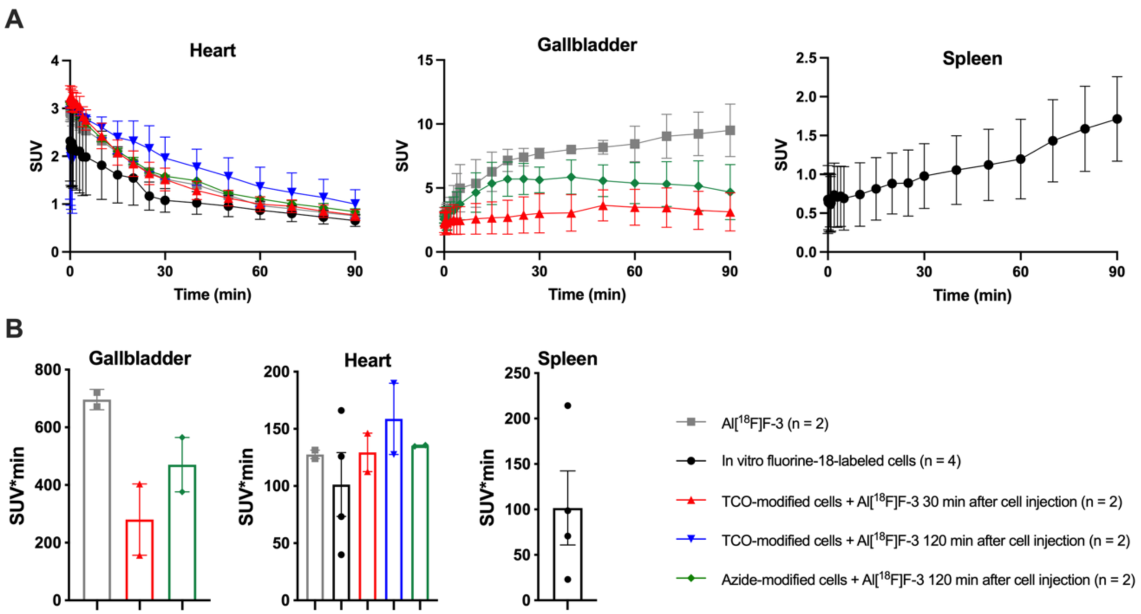

Figure S21. Additional TACs and AUCs. Quantitative PET analysis and ex vivo biodistribution of **Al[^{18}F]F-3**, in vitro fluorine-18-labeled cells, and pretargeting experiments (TCO-modified cells followed by **Al[^{18}F]F-3** injection after 30 min, TCO-modified cells followed by **Al[^{18}F]F-3** injection after 120 min, and azide-modified cells followed by **Al[^{18}F]F-3** injection after 120 min). **A**. The time-activity curves (TACs) were derived from dynamic PET imaging for selected ROIs. Data points correspond to mean±SEM (n = 2). **B**. Areas under the curve (AUC) analysis for selected ROIs. Error bars are represented by SEM.

Table S6. Statistical comparison of time–activity curves (TACs) between control (**Al[^{18}F]F-3**) and cell-based tracers using repeated measures two-way ANOVA with Šídák’s multiple comparisons test. Reported are the p-values for the time × group interaction (time × column factor), indicating whether TAC profiles differed over time between groups.

	Al[^{18}F]F-3 (n = 2)	In vitro fluorine-18-labeled cells (n = 4)	TCO-modified cells + **Al[^{18}F]F-3** 30 min after cell injection (n = 2)	TCO-modified cells + **Al[^{18}F]F-3** 120 min after cell injection (n = 2)	Azide-modified cells + **Al[^{18}F]F-3** 120 min after cell injection (n = 2)
Urine		ns (p=0.1477)	ns (n=0.2399)	ns (p=0.4106)	ns (p=0.3061)
Gallbladder		-	ns (p=0.2243)	-	ns (p=0.3747)
Liver		*** (p=0.0002)	ns (p=0.5554)	ns (p=0.4479)	ns (p=0.6467)
Kidney	Control	* (p=0.0191)	* (p=0.0315)	* (p=0.0243)	ns (p=0.1009)
Heart		ns (p=0.6713)	ns (p=0.3144)	ns (p=0.4628)	ns (p=0.7117)
Lung		ns (p = 0.0558)	ns (p=0.7764)	ns (p=0.4395)	ns (p=0.1858)
Small intestine		** (p=0.0093)	ns (p=0.3439)	ns (p=0.2304)	ns (p=0.2347)

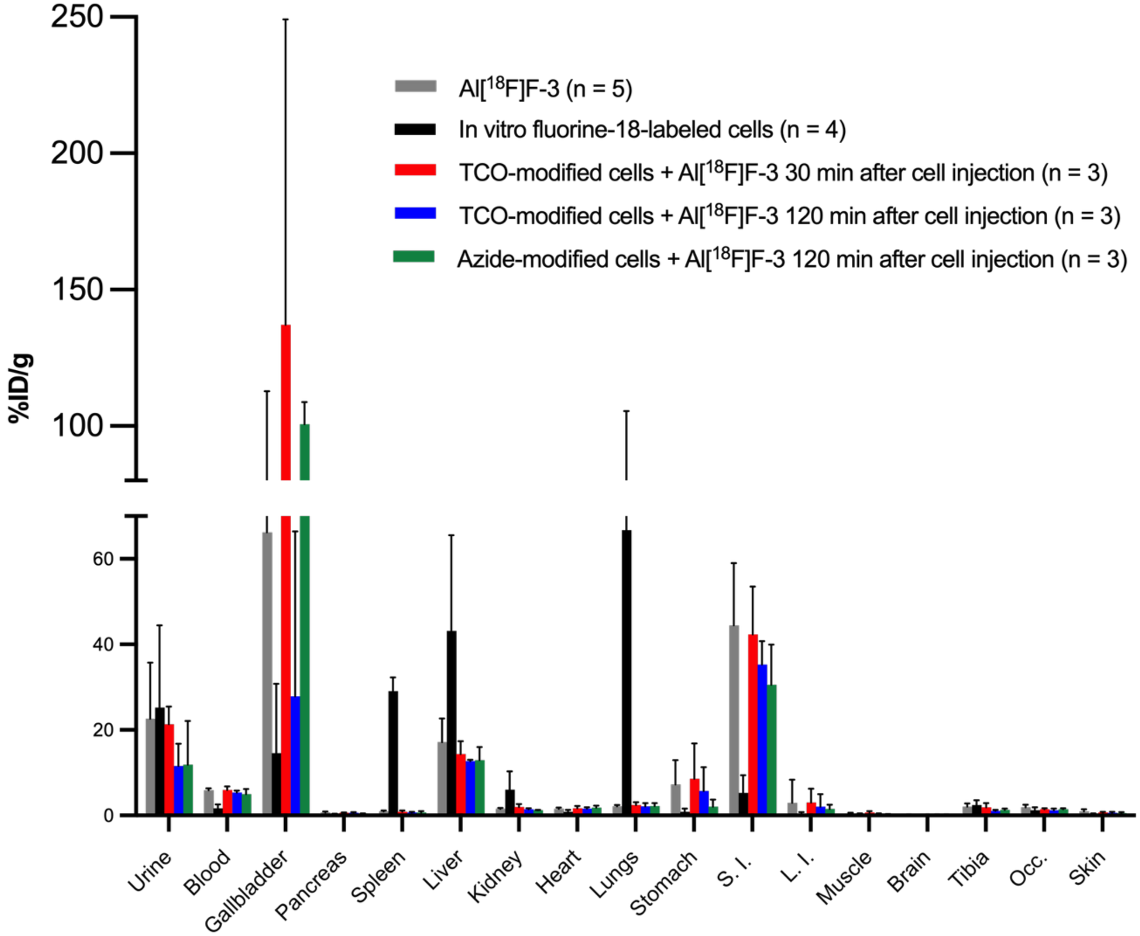

Figure S22. Complete ex vivo biodistribution of **Al[^{18}F]F-3,** in vitro fluorine-18-labeled cells, and pretargeting experiments (TCO-modified cells followed by **Al[^{18}F]F-3** injection after 30 min, TCO-modified cells followed by **Al[^{18}F]F-3** injection after 120 min, and azide-modified cells followed by **Al[^{18}F]F-3** injection after 120 min). Results are presented as percent injected dose per gram of tissue (%ID/g, mean±SD, n = 3–5).

Table S7. Ex vivo biodistribution values after a 90-min PET/CT dynamic scan. Values represent %ID/g, mean±SD.

	**Al[^{18}F]F-3** (n = 5)	in vitro fluorine-18-labeled cells (n = 4)	TCO-modified cells + **Al[^{18}F]F-3** 30 min after cell injection (n = 3)	TCO-modified cells + **Al[^{18}F]F-3** 120 min after cell injection (n = 3)	Azide-modified cells + **Al[^{18}F]F-3** 120 min after cell injection (n = 3)
Urine	22.63±13.1	25.26±19.16	21.33±4.12	11.62±5.13	11.89±10.24
Blood	5.90±0.46	1.65±0.96	5.94±0.86	5.38±0.40	4.99±1.16
Gallbladder	66.19±6.59	14.60±16.19	137.12±112.06	27.86±38.62	100.69±8.07
Pancreas	0.70±0.25	0.24±0.18	0.61±0.07	0.62±0.17	0.45±0.04
Spleen	0.94±0.21	29.1±3.19	0.87±0.30	0.71±0.16	0.71±0.27
Liver	17.16±5.53	43.14±22.39	14.37±2.98	12.73±0.28	12.94±3.10
Kidney	1.57±0.18	6.07±4.27	2.02±0.63	1.45±0.26	1.25±0.06
Heart	1.54±0.29	0.88±0.44	1.7±0.50	1.65±0.31	1.81±0.51
Lung	2.15±0.30	66.74±38.77	2.47±0.65	2.13±0.74	2.22±0.65
Stomach	7.22±5.70	0.86±0.78	8.58±8.25	5.76±5.51	2.04±1.65
Small intestine	44.47±4.52	5.28±4.11	42.32±11.18	35.29±5.48	30.58±9.36
Large intestine	2.96±5.40	0.44±0.38	3.04±3.18	2.06±2.95	1.52±0.96
Muscle	0.50±0.15	0.25±0.26	0.73±0.31	0.40±0.10	0.31±0.04
Brain	0.19±0.05	0.09±0.04	0.16±0.03	0.18±0.07	0.19±0.10
Tibia	2.08±0.74	2.41±1.17	1.90±1.02	1.12±0.19	1.3±0.33
Occipital	1.90±0.6	1.20±0.72	1.47±0.21	1.25±0.37	1.48±0.20
Skin	0.93±0.5	0.27±0.19	0.70±0.14	0.66±0.18	0.56±0.23

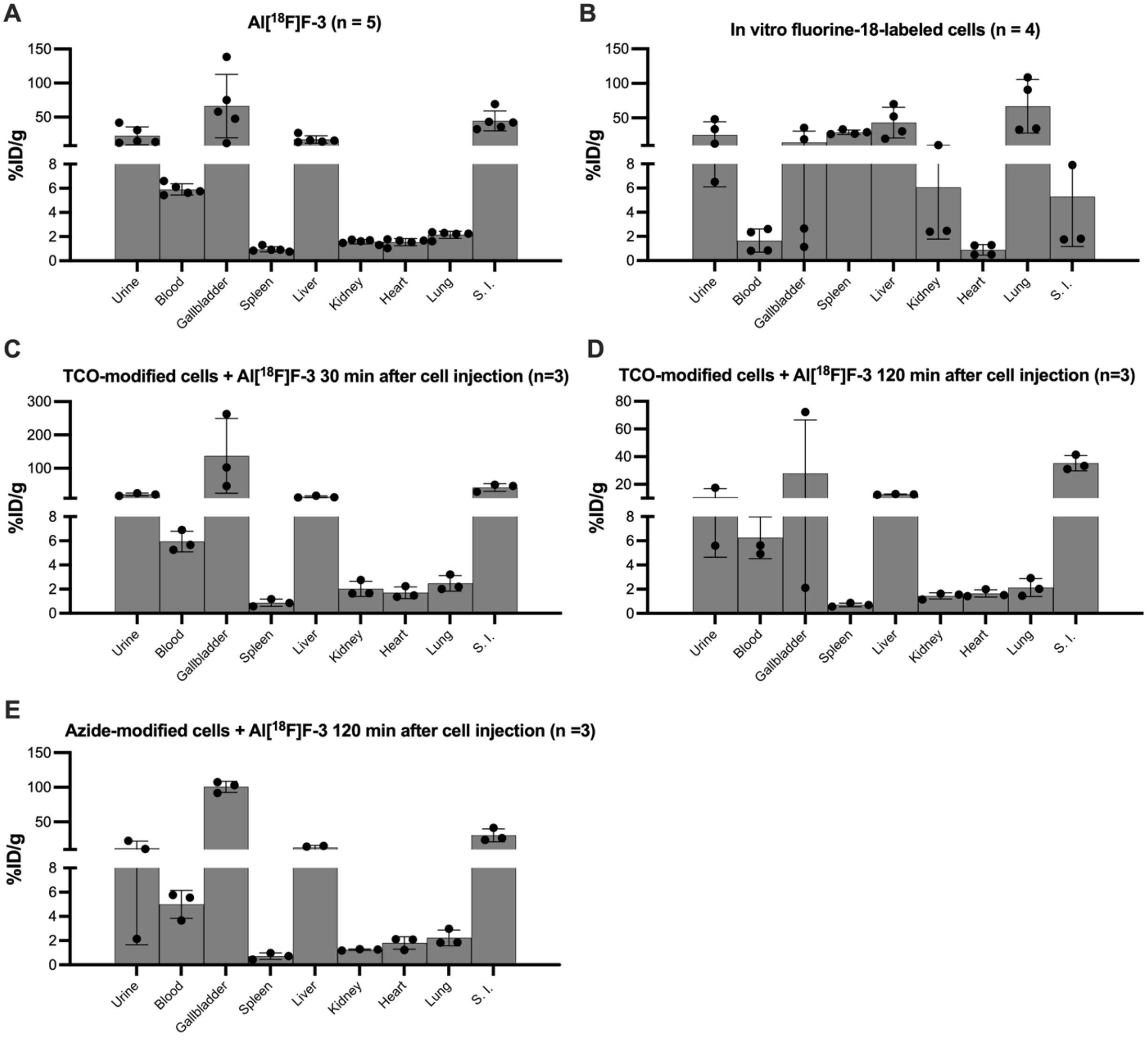

Figure S23. Ex vivo biodistribution in selected organs per condition. **Al[^{18}F]F-3 (A),** in vitro fluorine-18-labeled cells (**B**), and pretargeting experiments (TCO-modified cells followed by **Al[^{18}F]F-3** injection after 30 min in **C**, TCO-modified cells followed by **Al[^{18}F]F-3** injection after 120 min in **D**, and azide-modified cells followed by **Al[^{18}F]F-3** injection after 120 min in **E**). Results are presented as percent injected dose per gram of tissue (%ID/g, mean±SD, n = 3–5).

Statistical methods

Data are represented as mean±SD for experiments done in replicate experiments (n > 1) except for TACs and AUCs, where the data points correspond to mean±SEM (n = 2). All data pertaining to the in vivo evaluation were compared to the control **Al[^{18}F]F-3.** TACs were analysed using repeated measures two-way ANOVA with Šídák's multiple comparisons test, whereas AUC values and ex vivo biodistribution data were compared using the Mann-Whitney U test. Statistical significance (*p*-value) thresholds were set at $^{*}p < 0.05$, $^{**}p < 0.01$, $^{***}p < 0.001$, and ns denoting not significant. GraphPad Prism 10 (version 10.6.1) software was used for statistical analysis and graphical representation.
