# Supplementary figures and images for: Metabolic Glycoengineering Enables Fluorine-18 Radiolabeling of T Lymphocytes via Dual-Bioorthogonal Chemistry
Source: Bioconjug Chem. 2026 Apr 24;37(5):981–94. doi: 10.1021/acs.bioconjchem.6c00052 (PMC13195574; doi:10.1021/acs.bioconjchem.6c00052)

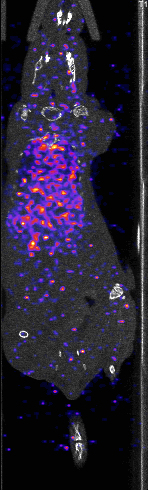

Supplement: Supplementary file 2 [file bc6c00052_si_008.gif]

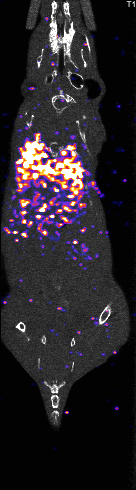

Supplement: Supplementary file 3 [file bc6c00052_si_009.gif]

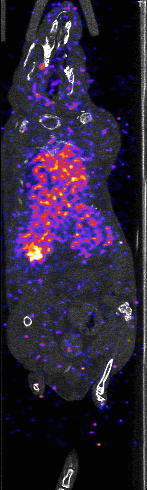

Supplement: Supplementary file 4 [file bc6c00052_si_010.gif]

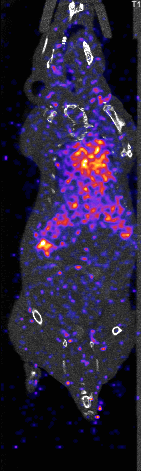

Supplement: Supplementary file 5 [file bc6c00052_si_011.gif]

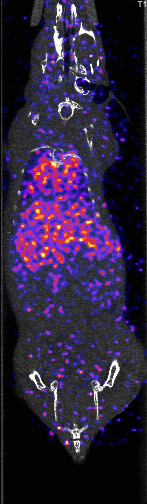

Supplement: Supplementary file 6 [file bc6c00052_si_012.gif]
